# Active Screening for *C. auris* Colonisation: A Preventive Step or an Excessive Measure? A Systematic Review

**DOI:** 10.3390/jof12080603

**Published:** 2026-08-13

**Authors:** Margarida Alves, Elisabete Ricardo, Sofia Costa de Oliveira

**Affiliations:** 1 Faculty of Medicine, University of Porto, Alameda Prof. Hernani Monteiro, 4200-319 Porto, Portugal; margaridatalves02@gmail.com; 2 RISE-Health, Faculty of Medicine, University of Porto, Alameda Prof. Hernani Monteiro, 4200-319 Porto, Portugal; ericardo@med.up.pt

**Keywords:** colonisation, screening, *Candida auris*, *Candidozyma auris*, surveillance, infection control

## Abstract

Background: Patients’ and surfaces’ colonisation play a pivotal role in *C. auris* transmission. Understanding how, when, and where screening for colonisation should be done is important to control the spread. Methods: This systematic review searched studies regarding *C. auris* patient colonisation, without previous outbreaks, until October 2024. Patient demographic and clinical data, sample type, collecting procedure, laboratory methods for strain identification, Clade determination, and susceptibility profile were collected. Results: Thirty-eight studies published between 2019 and 2023 across 18 countries were included using well-established inclusion criteria. Various body sites were screened mainly in patients admitted to intensive care units (ICUs). Patients had various comorbidities and were more commonly colonised during hospitalisation, compared to admission. Most of the isolates belonged to Clade I. Of 19 studies determining antifungal susceptibility, 15 reported resistance, mainly to azoles. Azole resistance was consistently associated with *ERG*11 substitutions (Y132F/K143R in Clade I, and V125A/F126L in Clade III). Conclusions: Healthcare workers should be on high alert for older, long-term hospitalised, ICU patients with comorbidities and co-colonisation with other pathogens. Composite bilateral axilla-groin sampling should remain the standard minimum approach. Additional anatomical sites may be considered. Well-equipped laboratories are vital for precision identification and antifungal susceptibility testing.

## 1. Background

*Candidozyma auris* (*C. auris*), previously known as *Candida auris*, is a multidrug-resistant yeast that was first isolated in 2009 from a patient’s ear discharge in Japan [1]. Since then, *C. auris* has been exponentially linked to severe infection all over the globe, being responsible for outbreaks in more than 50 countries [2]. With mortality rates ranging between 29% and 62% [3], this yeast is now considered by the World Health Organisation (WHO) a critical priority fungal pathogen [4].

The concern surrounding this yeast starts with its ability to spread, colonise, and have high transmissibility, therefore being able to cause outbreaks [5,6]. These characteristics are related to its ability to persist on medical devices, surfaces, and human skin [7], which facilitates transmission. It can occur via infected individuals or contaminated surfaces, such as those frequently touched by patients or healthcare workers (e.g., beds, medical devices), as well as areas out of patients’ reach (e.g., air vents, computers, and medical carts) [5,8]. In addition, *C. auris* can cause invasive infections, such as bloodstream infections, meningitis, endocarditis, and intra-abdominal infections [7].

This emergent yeast can asymptomatically colonise the axillae, groin, nares, respiratory and urinary tracts [5], which can progress, in nearly 10% of *C. auris*-colonised patients, to invasive infections, particularly those under mechanical ventilation and with indwelling medical devices, admitted to intensive care unit (ICU) settings [2].

There are different identified risk factors predisposing to infection by *C. auris*, such as corticosteroid therapy, a prolonged stay in ICU, mechanical ventilation, presence of a central venous catheter or urinary catheter, prolonged exposure to broad-spectrum antibiotics or antifungal use, or surgery [5].

To date, six different clades have been identified, namely the South Asian (I), East Asian (II), South African (III), South American (IV), a more recently reported Iranian clade (V) and lately a sixth clade identified first in Singapore and recently in Bangladesh (VI) [9,10,11,12]. The antifungal susceptibility profile of *C. auris* is particularly alarming, with strains exhibiting high minimum inhibitory concentrations (MICs) for azoles and a resistance phenotype to echinocandins and amphotericin B [9,13,14], which are more common in some clades than others [5,7]. High MIC values for fluconazole are the most frequent, being around 90% in the United States of America (USA), including newer azoles. For other antifungal agents, such as amphotericin B, the numbers decrease to around 30%, with resistance to echinocandins being the rarest, at 3–7% [9,15]. Although uncommon, the emergence of resistance to echinocandins is concerning, as this class is the recommended first-line therapy for *C. auris* infections [5].

The colonisation of patients and surfaces by *C. auris* plays a pivotal role in its transmission dynamics, with colonisation often preceding infection [16,17]. The skin microbiota serves as a critical reservoir for *C. auris*, with interactions between *C. auris* and the skin microbiota complex influencing both the pathogen’s persistence on the host and its transmission capacity. Understanding these dynamics is crucial for developing effective infection-control and prevention strategies, yet gaps remain in our knowledge regarding the specific mechanisms facilitating *C. auris* spread from colonised patients to the environment and subsequently to other patients [18,19,20]. Epidemiological studies have reported colonisation rates ranging from 5% to 70% amongst ICU patients, depending on the geographic location, patient population, and local infection control practices [17,21,22]. Since 2022, there has been a substantial increase in cases in European Union/European Economic Area (EU/EEA) countries, with some countries stating that it was no longer possible to distinguish between an outbreak and national endemicity, highlighting the potential of this yeast to spread [23]. Despite the increase already mentioned, active surveillance is only present in less than half of the EU/EEA countries, which means that the real numbers are underestimated [23].

This systematic review aims to clarify whether screening of *C. auris* colonisation is justified and specify how, when, and where it should be performed.

## 2. Material and Methods

The systematic review was carried out in strict accordance with the Cochrane Handbook for Systematic Reviews of Interventions [24], and the Preferred Reporting Items for Systematic Reviews and Meta-Analyses (PRISMA) [25] checklist was followed (Supplementary Materials—Table S1).

### 2.1. Data Source and Search Strategy

The electronic bibliographic databases of PubMed, Scopus and Web of Science were searched using a combination of MeSH terms and keywords: (colonization OR colonisation) AND (“Candida auris” OR “C. auris”). Studies were selected and screened until October 2024. The search included all publication types except reviews or systematic reviews and editorials, and no language restrictions were applied.

### 2.2. Eligibility Criteria

Only studies addressing *C. auris* patient colonisation without reports of previous outbreaks or not primarily designed as outbreak investigations were included in this systematic review. Colonisation with *C. auris* was defined as the presence of *C. auris* in human biological samples without signs or symptoms of infection. Studies that reported only *Candida auris* management, treatment, laboratory diagnostics, susceptibility, mechanisms of antifungal resistance, and environmental screening were excluded. Studies exclusively related to hospital disinfection procedures or to surface decontamination efficacy were excluded, as were systematic reviews, editorials, and grey literature.

### 2.3. Data Extraction, Synthesis, and Analysis

**Figure 1:**
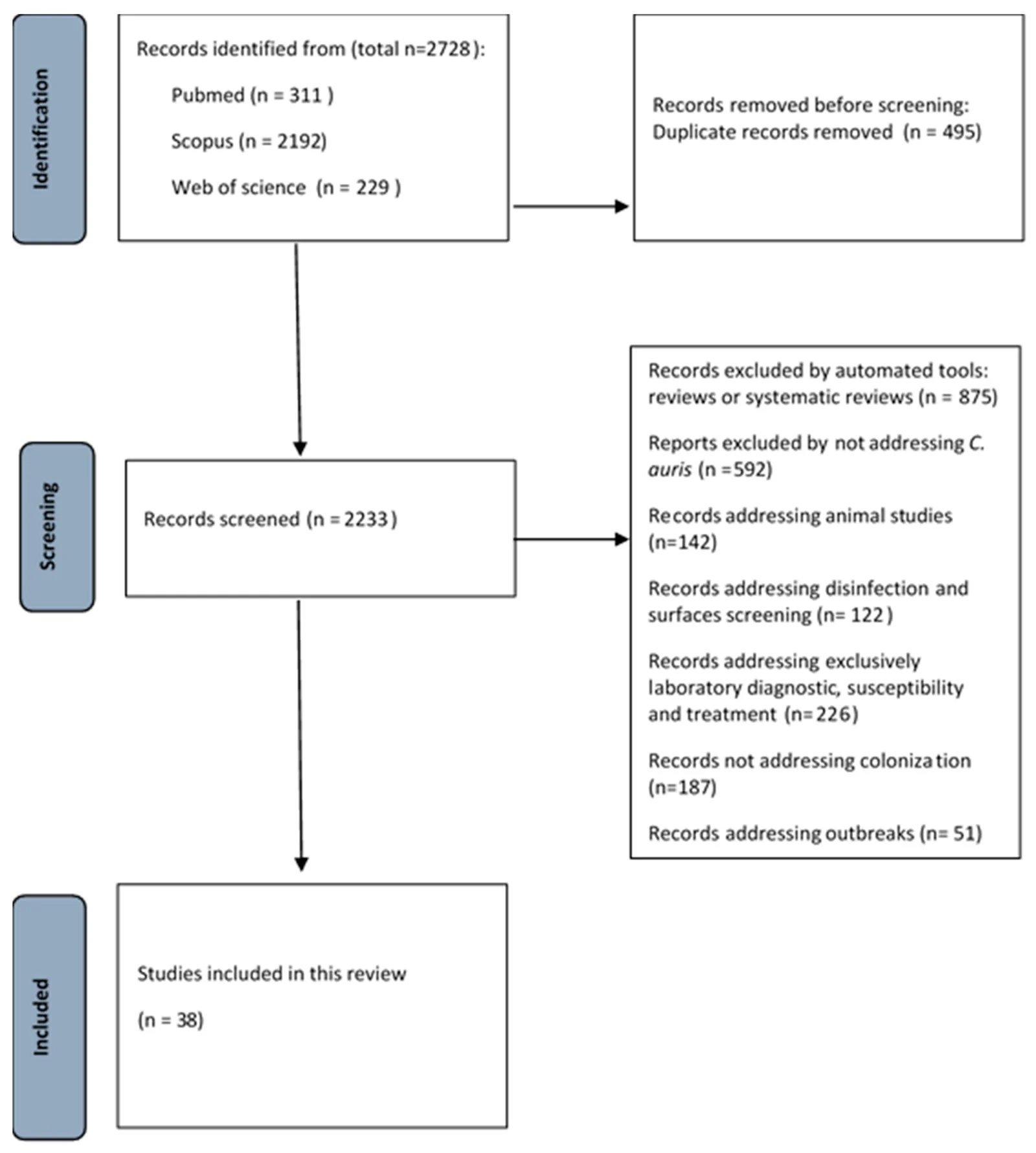
PRISMA flow chart representing the systematic identification of studies searched via databases and registers.

A total of 2728 references were obtained in the three databases. The literature screening followed a 2-step process. The second phase involved reviewing the full text of the selected studies. Disagreements were resolved by consensus. The PRISMA flowchart (Figure 1) summarises the literature search and screening strategies. The extracted studies were uploaded to EndNote and the Rayyan software (Version 4.1.3.) [26] for duplicate removal and further selection. In a blinded, standardised manner, a screening of the title, abstract, and full text was performed and guided by two independent reviewers (MA and SCO) based on inclusion and exclusion criteria. Thirty-eight studies were included in the systematic review (Figure 1). A protocol was developed to uniformly and consistently synthesise the data collected from the selected studies. Data such as year of publication, country, patient demographic and clinical data, sample type, collecting procedure, laboratory methods for strain identification, Clade determination, and susceptibility profile were extracted from the included publications. MA and SCO independently extracted the above information. No meta-analysis was performed due to the heterogeneity of the studies.

### 2.4. Risk of Bias (RoB) Assessment

Risk of bias was assessed by SCO and ER using the Joanna Briggs Institute (JBI) Critical Appraisal Checklists [27] because the included evidence comprised a heterogeneous set of non-randomised study designs. Specifically, the JBI checklists were applied for cohort studies, case–control studies, analytical cross-sectional/prevalence studies, case series, and case reports, as appropriate. Each checklist item was judged as Yes, No, Unclear, or Not applicable, based on information reported in the article. For synthesis, item-level judgments were converted into an overall RoB category using a predefined rule: studies were classified as low RoB when all key items were met and there were no major concerns, moderate RoB when one or more important items were Unclear or No but not sufficient to seriously compromise internal validity, and high RoB when multiple key items were No/Unclear.

## 3. Results

In this review, 38 studies were analysed, with publication years ranging from 2019 to 2024, mainly in 2021; the study period/collection of samples spanned between 2010 and 2023.

### 3.1. Characteristics of the Selected Studies

**Figure 2:**
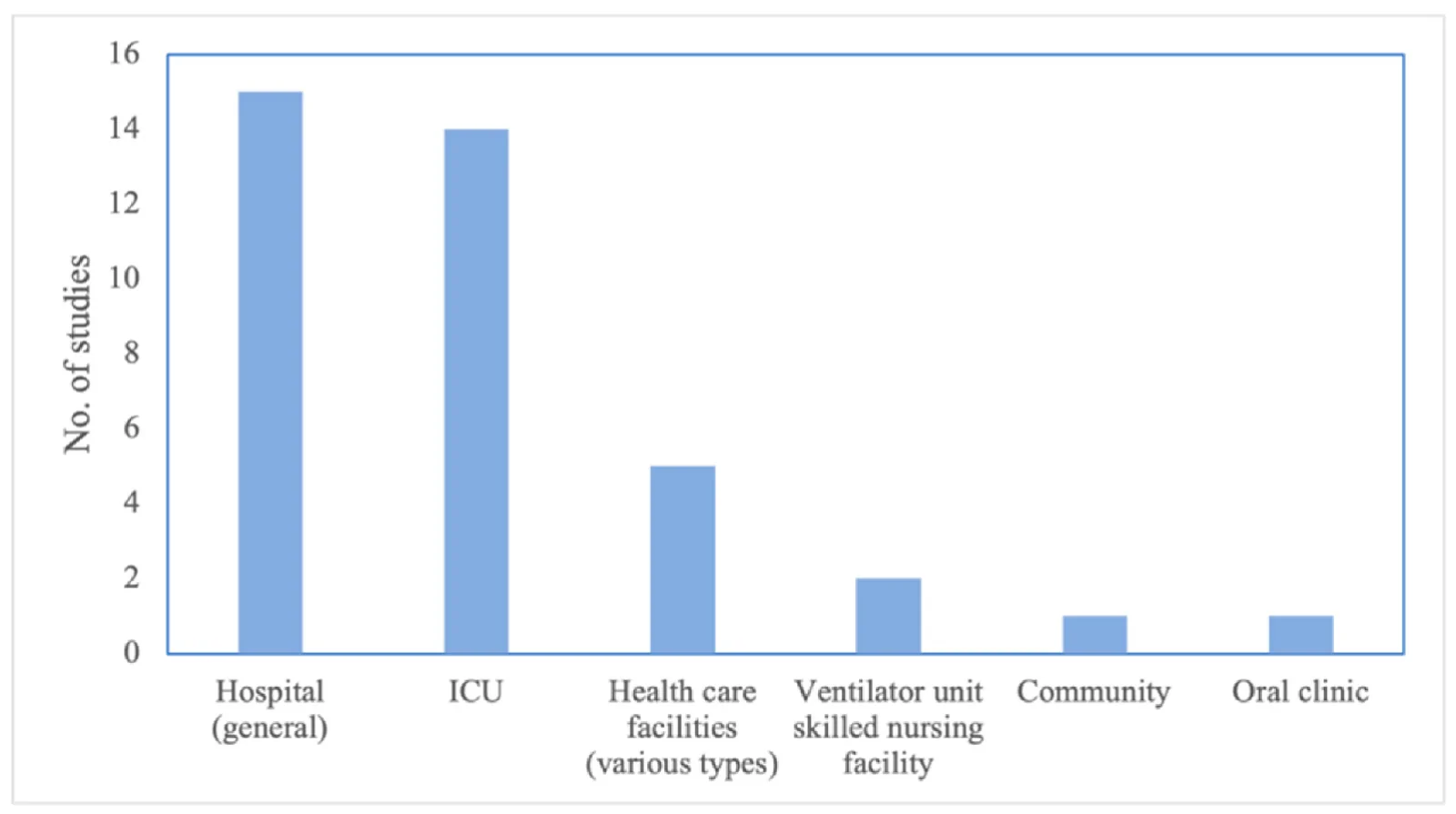
Distribution of studies according to health care facility or community settings.

The studies were from 18 countries, comprising observational studies from North America and Europe and additional reports from the Middle East, Asia and South America (Table 1). Most studies were conducted in hospital wards and ICUs (Figure 2). Five studies included patients from different healthcare facilities, with one of them studying patients in the custody of a state or federal prison [28]. Only one study had patients in a community setting [29] and one was conducted in an oral clinic [30].

Reported colonisation burden varied markedly by setting: point prevalence and admission screening studies in ventilator-capable facilities and nursing homes often showed the highest proportions (including very high facility-level prevalence), whereas several hospital-based screening efforts reported low prevalence or no detected colonisation (including studies with zero positives) (Table 1). Particularly, three studies did not find any cases of *C. auris* [30,31,32]. One was conducted in an oral clinic on saliva samples [30], the other two were both conducted in an ICU, with one using a composite bilateral axillary/inguinal swab, and the other swabs from nose, throat, axillae, groin, perineum, rectum and a catheter urine sample [31,32]. Sharp et al. used specifically hospitals with no known history of *C. auris* in order to guarantee that the cases detected were imported from the community, with an approach more focused on local epidemiology [32].

Most studies (n = 16) found the cases during hospitalisation, ranging from 1 week to several months. One study stated that 35.1% of patients tested negative at least once before testing positive [33]. Eight studies found colonised patients during admission [33,34,35,36,37,38,39,40], one having 0.8% of patients colonised on admission, with an increase to 16.8% on week four of hospitalisation [38]. Table 1 also highlights that, besides a colonised preterm baby of 25 weeks of gestational age [34], colonisation of *C. auris* only occurred in adults, mainly in middle-aged adults between 60 and 70 years, followed by the age range of 70–80 years. Cases in younger ages occurred but were less common, mainly below 50 years. This was seen in two studies that compared age ranges, with 56–75 years [41] and 71–80 years [42] having the highest number of cases.

### 3.2. Risk Factors and Clinical Outcome

The majority of the reviewed studies reported that colonised patients were not treated with antifungal drugs. In Mesini et al., the patient had received fluconazole prophylaxis, a standard for low-birth-weight neonates [34]. One study, focused on the decolonisation of *C. auris*, reported that colonised patients were either decolonised with chlorhexidine with concomitant antifungals or concomitant antibiotics and antifungal [43]. Table 2 demonstrates frequent *C. auris* co-colonisation with other pathogenic organisms, the most common carbapenem-resistant organisms (CRO), followed by multidrug-resistant organisms (MDRO) such as methicillin-resistant *Staphylococcus aureus* (MRSA), vancomycin-resistant *Enterococci* (VRE) and extended-spectrum beta-lactamase (ESBL) producing *Enterobacterales*. One study in particular found a statistically significant difference in the presence of MDRO in the *C. auris* colonised group compared to the non-colonised group [44]. Other *Candida* spp, such as *C*. *albicans*, *C. tropicallis*, *C. parapsilosis* and *C. glabrata,* were co-colonisers in seven different studies [35,40,45,46,47,48,49], as well as *SARS-CoV-2* in three studies [50,51,52]. *Proctor* et al. found that *C. auris* colonised samples were enriched in specific bacteria such as *Proteus mirabilis*, *Klebsiella pneumoniae*, *Providencia stuartii*, and *Pseudomonas aeruginosa* [53].

The progression from *C. auris* colonisation to invasive infection was documented in thirteen studies, most often as bloodstream infection (BSI) but also as urinary tract infection (UTI), septic arthritis, and other invasive presentations. *C. auris*, compared to other species, had a higher probability of fungemia in colonised patients (12.32%), and a higher risk of developing fungemia associated with a higher number of colonised body sites [38], being an infection frequently preceded by patient colonisation. In contrast, eleven studies reported no development of infection amongst colonised patients. One study documented that one of the infected patients died; however death was possibly related to carbapenem-resistant *Pseudomonas aeruginosa* infection [50], in another, 30-day mortality after the onset of *C. auris* candidaemia was 26% [51]. In contrast, in another study none of the infected patients died [37]. One study showed a statistically significant association between pharyngeal colonisation and a positive blood culture [54], with another stating that tracheal/bronchial colonisation had a high positive predictive value (32.84%) for predicting subsequent fungemia, followed by urine colonisation (25.44%) [38].

Mortality in colonised patients was addressed in 16 studies. In most studies, the cause of death was unknown or not specified, with the attributable role of *C. auris* being uncertain, with only two studies stating that the death was unrelated to *C. auris* [29,36]. Of a total of three case reports that relay information about mortality, the patients died in all. Reported mortality varied widely by setting, ranging from 17.5% to 31% in a 30-day mortality rate, and from 16.7% to 37.7% in a 90-day mortality rate, especially in highly dependent populations. Rossow et al. showed that the 90-day mortality rate in colonised patients (16.7%) was significantly higher than in non-colonised patients [44]. Furthermore, colonisation with *C. auris* alone or with MDRO increased mortality risk in COVID-19 patients [52].

**Figure 3:**
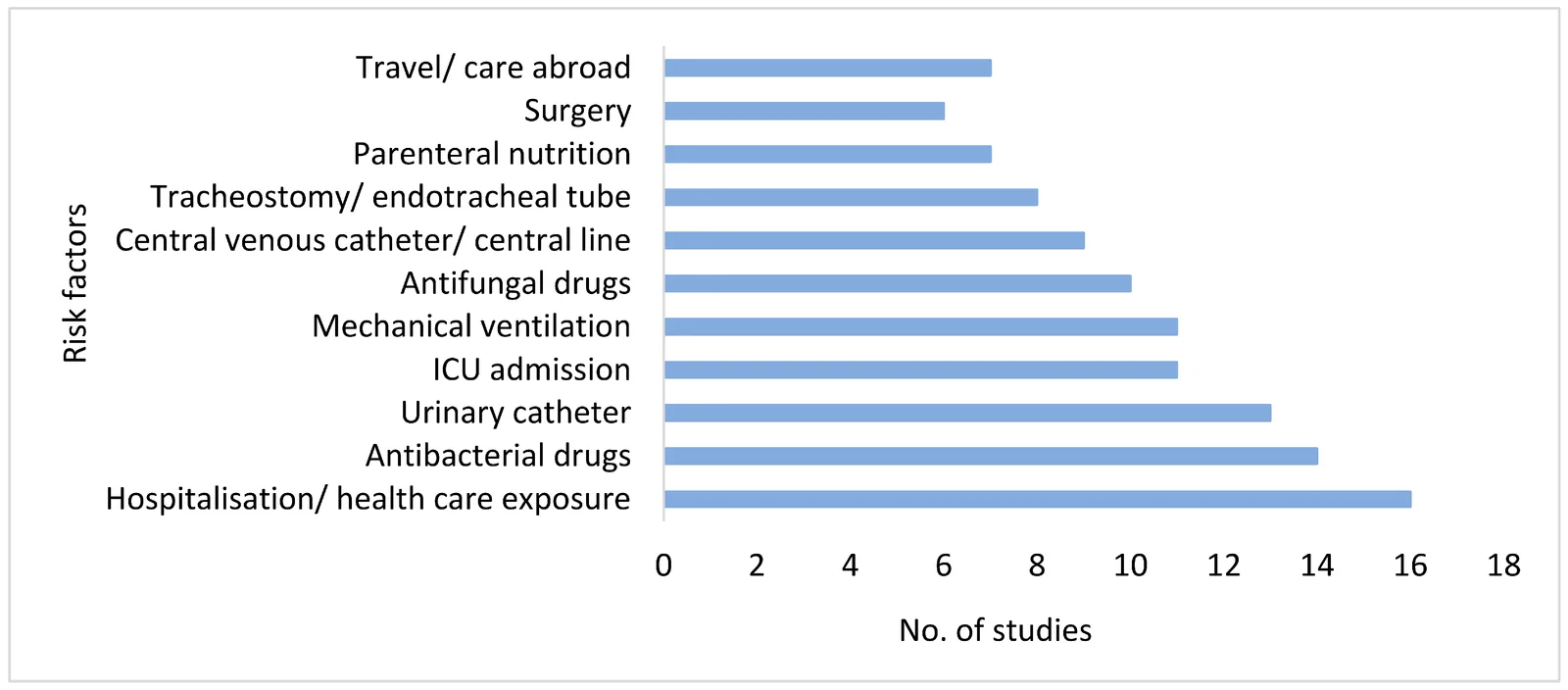
Distribution of studies according to most frequently reported previous exposures.

This review identified common risk exposures amongst patients (Figure 3). Previous surgery was reported in six studies, and a known history of travel or healthcare contact in a different country was present in seven studies, mainly in India, but also Saudi Arabia, Mauritius and the United Arab Emirates.

History of previous antibiotic treatment was more frequently reported across the included studies (n = 14) than exposure to antifungals (n = 10). Specifically, previous antibiotic exposure was significantly more common in colonised patients than in non-colonised patients in two studies [44,53]. Similarly, prior antifungal use was significantly more common in colonised patients in two studies [33,44]. However, other studies did not find these correlations [33,53].

Most studies reported that patients had previous contact with hospitals, either in acute care hospitals, inpatient medical care, or mainly through hospitalisation. Acute care visits were, in one study, significantly more common in colonised patients compared to non-colonised [44]. Most studies also had patients who had been in contact with ICUs, and some studies reported contact with long-term care facilities.

Urinary catheters were the most common medical device used in colonised patients, followed by mechanical ventilation and central venous catheter (CVC). A tracheostomy tube and a feeding tube were also common amongst patients. Mechanical ventilation, urinary catheters and feeding tubes were in two studies and CVC and drains in one study, significantly more common in colonised patients compared to non-colonised patients [33,44,53].

Tracheostomy was significantly more common in two studies, while *Proctor* et al. did not find this association [33,44,53].

### 3.3. Colonisation Sites, Sampling and Laboratory Diagnostics

**Figure 4:**
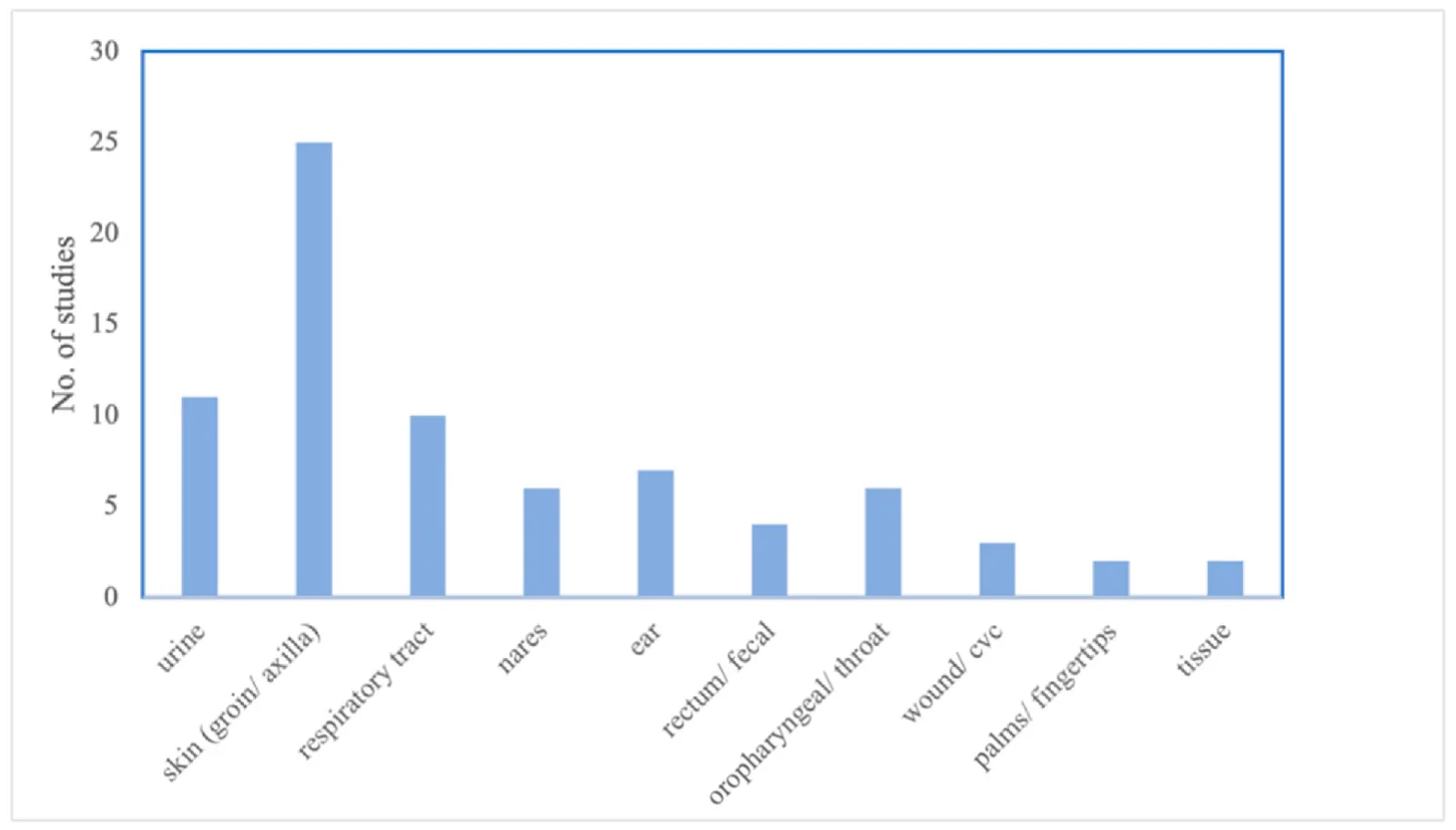
Distribution of studies with positive screening for *C. auris* according to body sites.

Patient colonisation was mostly detected through skin screening, particularly axillae and groin swabs, often complemented by additional body sites depending on setting and purpose (e.g., nares, rectum, oropharynx/throat) (Table 3 and Figure 4). Clinical samples, such as urine and from the respiratory tract, were frequent sources of detection in hospitalised and ventilated patients (with several reports highlighting urine, respiratory tract and broader skin positivity, and others emphasising rectal predominance). Screening approaches ranged from targeted composite swabbing (axillae/groin ± nares) to extensive multisite protocols in high-risk facilities.

For *C. auris* detection and identification, most studies (n = 25) relied on culture-based screening using Sabouraud dextrose agar (SDA) and/or chromogenic Candida media, with identification primarily performed using matrix-assisted laser desorption ionisation-time of flight mass spectrometry (MALDI-TOF MS). Three studies confirmed MALDI-TOF’s identification with sequencing of internal transcribed spacer regions (ITS) D1-D2 and one with whole-genome sequencing (WGS) [40,46,55,56].

Polymerase chain reaction (PCR) was used in 13 studies, three as the primary screening test, 10 alongside culture, and in one to identify any potential *Candida* cryptic species.

### 3.4. Phylogeny, Susceptibility Profile and Mechanisms of Antifungal Resistance

*C. auris* Clade was determined in 11 studies, with the most common the South Asian Clade (I) [35,39,40,42,47,55,57,58,59], followed by the South African Clade (III) [42,60] and the South American Clade (IV) (Table 4) [53].

Antifungal susceptibility testing (AST) across the included studies was performed in 17 studies [34,35,36,39,40,41,42,45,47,50,51,53,54,57,58,59,60] mainly using broth microdilution according to Clinical and Laboratory Standards Institute (CLSI) [35,36,39,41,42,51,57,58,60], one according to European Committee on Antimicrobial Susceptibility Testing (EUCAST) [47], with one study using both [35]. In some cohorts, automated systems such as Vitek 2 were used [45,50].

The MIC cutoffs were mainly based on the Centre for Disease Control and Prevention (CDC) tentative breakpoints [34,39,40,45,50,51,52,53,60].

Fluconazole high MIC values were frequently reported, reaching 100% in multiple reports [35,41,51] and remaining high (78.5%) in others [45]. Amphotericin B resistance showed marked variability between studies, between 7.9% and 56% [35,41,51,60], while echinocandins were reported as susceptible, though scarce non-susceptibility or resistance was described (anidulafungin resistance in some clade III isolates and caspofungin resistance) [42,58]. Azole resistance was consistently associated with *ERG*11 substitutions (Y132F and/or K143R in clade I studies, and clade III-associated variants such as V125A/F126L), with additional putative mechanisms including *TAC*1B substitutions and increased *MDR*1 copy number [35]. Regarding echinocandin resistance, an *FKS*1 hotspot-1 mutation (S639P) was found [58].

### 3.5. Risk of Bias—Quality Assessment

JBI critical appraisal checklists according to study design are detailed in Table S2 (Supplementary Materials). Briefly, 9 out of 38 studies were classified as low risk of bias, 24 as moderate risk of bias, and five as high risk of bias. Low RoB ratings were mainly observed in well-described cohorts with clearly defined eligibility criteria, laboratory confirmation, and detailed case reports, whereas moderate RoB predominated in retrospective cohorts, case–control studies, and case series, where key items were frequently Unclear, particularly complete inclusion, completeness of follow-up, and control of confounding variables. High RoB ratings were driven largely by selection concerns in screening or prevalence-type studies and by limited confounding management and outcome in some retrospective observational datasets.

## 4. Discussion

Given its high transmissibility in clinical settings, rapid detection of *C. auris* and immediate implementation of contact precautions are vital to halting its spread. The implementation of risk-based and response-based screening, rather than indiscriminate universal screening everywhere, is mandatory, with a well-established definition of where, how, when and who.

### 4.1. Where to Screen?

As recommended by the CDC, skin (axillae and groin composite swabs) was the most commonly screened body site, followed by urine and the respiratory tract [66]. According to this review, multisite colonisation was common [46,51,53]. Hence, screening multiple body sites increased sensitivity compared to single body sites [53,61], with the maximum sensitivity achieved by screening at least six body sites [53]. Another important issue to consider is bioburden. Besides axillae and groin, other body sites also have higher bioburdens, such as the nares and palms/fingertips, which are not routinely screened, and may contribute to outbreaks [53].

The CDC recommended composite bilateral axillae and groin swabs achieved a lower proportion of identification than adding the nares, yielding about 12–18% additional capture [53,61]. Palms/fingertips could also be a possible addition [53,61], according to Proctor et al., who found monocolonisation mainly in nares or palms/fingertips, with these places also having higher bioburdens [53]. In Lopez et al., a composite swab of anterior nares and hands improved sensitivity and was more consistent than axillae and groin [67]. The importance of screening the nares could also be because antiseptic bathing does not impact nasal carriage, suggesting that this body site might be related to outbreaks, and nasal decolonisation could be an important component for reducing *C. auris* carriage and preventing infection [61].

However, it is important to note that while culturing may yield a higher *C. auris* burden in the nares than in the groin, sequencing results may fail to identify it as the predominant species, due to the diversity, complexity, and overall bioburden of the nasal microbiome [53].

On the other hand, other studies showed that single area colonisation is more common, with the groin being the most monocolonised body site [35,43]. These studies tested only the groin and ear, with Yadav et al. also testing the nares, and Elbahr et al. the axillae [35,43]. In Yadav et al., no patients developed infection, which is in line with Garcia Bustos et al., who described that multifocal colonisation was observed to a greater extent in candidaemia patients [35,46]. Nobrega de Almeida et al. stated that no additional cases would have been missed if only screening of the axillae was performed; however, this finding was related to the high culture-positivity rate found for the digital thermometers [57].

This relationship between skin colonisation, particularly the number and bioburden of colonised body sites, and environmental contamination suggests that targeting this colonisation could be useful for controlling *C. auris* spread [61,62]. Therefore, screening different body sites from the axillae and groin could help reduce false negatives, because patients were frequently colonised at other body sites [53,67]. However, screening of a high number of body sites may not be practical for routine screening, highlighting the need to target sites colonised more frequently and with higher bioburdens [53]. These results suggest that screening axillae and groins with nares and/or palms/fingertips may be more effective for identifying colonised patients, possibly using a composite swab to reduce logistical challenges [67].

Multisite colonisation retained a significant association with the development of *C. auris* candidaemia [51]; the higher the number of colonised body sites, the higher the risk of developing fungemia [38]; therefore, knowing that a patient has multisite colonisation could help us identify patients at higher risk for infection [46].

*C. auris* not only colonises but also has a high invasive potential, particularly for bloodstream infection. Candidaemia was significantly more common in patients with multisite colonisation [46,51], with a recurrence rate of 26% [51]. When screening fails to identify colonisation, it leaves high-risk patients unidentified until invasive infection manifests, with candidaemia being the main clinical manifestation [63], highlighting the risk of a screening that does not identify the maximum number of colonised patients.

Therefore, colonisation should not be overlooked as a risk factor for developing bloodstream infection, since it also increases the risk of recurrence. Screening could help identify patients at risk in a timely manner, preventing the first manifestation of this yeast from being invasive candidaemia. All of this highlights the need for screening, because knowing the patient is colonised is knowing that they are at risk and therefore, we can try to prevent worse outcomes.

Colonisation of anterior nares and palms and/or fingertips suggests that nasal decolonisation and promotion of resident hand hygiene may be options to help reduce transmission [53].

After the skin, the most colonised body sites were urine and the respiratory tract. In Sayeed et al., the majority of colonised urine samples were from catheterised patients, with urinary catheters also being very common among colonised patients in general. Mechanical ventilation was also very common [41]. However, less than half of the studies with positive urine samples had a urinary catheter documented. The same was seen in respiratory tract samples.

One study in this review showed a statistically significant association between pharyngeal colonisation and a positive blood culture, finding no significant correlations between positive blood culture and skin colonisation [54]. In another, tracheal/bronchial colonisation had a high positive predictive value (32.84%) for predicting subsequent fungemia, followed by urine colonisation (25.44%) [38]. According to Park et al., amongst infected patients previously colonised, the respiratory tract was the most isolated site [45]. The question remains whether screening body sites with a positive association with the development of infection, followed by early antifungal therapy, particularly in critical units, could be of use to prevent infection, but more research is needed [38].

For now, according to current guidelines, using systemic antifungal treatment for colonisation is not advised because of the risk of promoting antifungal resistance; consequently, few included studies assessed this topic, leaving this question unanswered in this review. Furthermore, routine decolonisation (e.g., chlorhexidine bathing) is not recommended due to insufficient evidence; however, there are already some studies on this topic [61,66]. Elbahr et al. studied chlorhexidine bathing but did not achieve satisfactory results in multicolonised patients, in contrast with in vitro results, mainly because it only decreases the skin burden but does not fully eradicate the organism [68,69].

Focus on preventing transmission of *C. auris* remains within traditional infection-prevention strategies, including meticulous hand hygiene, isolating colonised or infected patients with contact precautions, personal protective equipment (e.g., gowns, gloves), decontamination of the environment and interfacility communication [40,61,64,66]. Nevertheless, the high number of positive urine and respiratory tract samples should not be overlooked, particularly given their association with fungemia. Therefore, passive screening should be maintained if clinically relevant or indicated in patients’ routine fungal culture of clinical specimens, particularly in urine and the respiratory tract [60].

It is also important to mention that in two studies *C. auris* predominantly colonised the rectum, having a high sensitivity for developing fungemia after colonisation [38,46]. Proctor et al. also stated that adding perianal skin to the screening could increase sensitivity, with this being the fourth most colonised body site [53]. Therefore, with MDRO screening already in place using swabs from the rectum, adding screening for *C. auris* in these swabs could be an easy way to increase the sensitivity of the screening [70,71].

### 4.2. How to Detect?

As previously stated, MALDI-TOF MS was the most commonly used method for *C. auris* identification, followed by PCR. Culture alone for identification was only used in two studies because, even though less expensive and easy to implement, the process is time-consuming due to the yeast growth being slow; it is also not easily distinguishable from other *Candida* spp. on standard agar plates, possibly missing colonies of *C. auris* [60,63].

Molecular methods, such as PCR or WGS, may have higher costs but have a higher sensitivity, can grant faster and more reliable and definitive results, detecting lower burdens of *C. auris* colonisation [63].

Thereby, culture, mainly without enrichment broths, has a low sensitivity compared to PCR [60]. The sensitivity of screening culture for *C. auris* may be dependent on the severity of illness, length of ICU stay or exposure to antimicrobials, being possible to increase the sensitivity by adding an enrichment broth step [32,72]. Compared to culture, PCR had a positive predictive value (PPV) of 91.4% in Bergeron et al. and 85.7% in Taori et al., with sensitivity and negative predictive values (NPV) of 100% and specificity of 99.4% [29,42].

Recent studies corroborate these results, reporting also a 100% NPV and a high sensitivity, highlighting the benefits of using these molecular tests for timely decisions, reducing hospital costs and shorter isolation periods, possibly justifying the higher cost [73,74].

MALDI-TOF, although time-consuming due to the need for prior culture, is highly selective with updated databases and can identify *C. auris* with high confidence. However, the need for prior culture, enriched media and incubation temperatures specific for *C. auris* for all screened samples makes it more time-consuming, compared to PCR, which is more efficient [72,75]. MALDI-TOF MS in the future could also be used for clade assessment and antifungal resistance testing, with spectral variation between clades, faster than WGS [76].

Therefore, PCR could be used for screening in order to reduce turnaround time, providing a closer-to-real-time assessment of a patient’s *C. auris* status, allowing rapid containment of transmission with optimum use of resources [42,62] and providing quick discontinuation of contact isolation precautions [63]. The observed negative predictive value of 100% provides confidence that a negative result excludes *C. auris* colonisation, a finding with relevant implications for screening. Sensitivity could even be enhanced by repeating PCR with a new swab rather than repeating PCR on the original swab, when results are indeterminate [60]. However, it is important to note that the meaning of a PCR-positive but culture-negative sample is uncertain because it may represent a nonviable organism [29], highlighting the importance of knowing the previous colonisation status of the patients. Also, PCR isolates cannot be used for antifungal susceptibility testing or outbreak tracing via whole-genome sequencing if necessary [73]. For these reasons, PCR could be used as a first assessment screening, but the need for characterisation of outbreaks, clade and antifungal susceptibility highlights the important role of culture and other methods such as MALDI-TOF and WGS.

Taori et al. also found a potential link between the Cycle Threshold (Ct) value of the qPCR result from patient screening swabs and the bioburden detected on the settle plates, which may provide a means of quantifying infectivity by PCR [42]. Some studies have also demonstrated that the Ct value in qPCR is related to the burden and growth of microorganisms for *SARS-CoV-2* and for M. Tuberculosis, with a low Ct value being able to identify a high burden, but more studies are needed for this conclusion [77,78].

### 4.3. When to Screen?

As previously stated, most patients were found to be colonised during hospitalisation, with an increase in colonised patients compared to admission. As seen in Biswal et al., *C. auris* is most likely acquired nosocomially [16].

A negative test result does not assure that the patient is not colonised, with intermittent positive and negative results being common and sustained negative results rare [37,62]. However, a single negative test should not be a criterion for discontinuation of transmission precautions [62]; testing multiple sites together with two distinct negative assessments could give us more certainty in the non-colonisation [29]. As seen in Lopez et al., screening different body sites from axillae and groin could help reduce false negatives [67]. Nonetheless, Pacilli et al. considered that rescreening patients is of limited use for infection control if there is no improvement in clinical status that could promote decolonisation [62], and Arenas et al. emphasise concerns about using negative test results as criteria for patient transfer or removing contact precautions [37]. As of right now, there are no clearance criteria for *C. auris* colonisation, mainly because of temporal inconsistencies in screening results. If possible, to improve the consistency of screening, criteria can be developed to determine decolonisation [66,67].

False negative results could represent colonisation below the detection limit, variations in sampling methods, intermittent shedding, and recolonisation after re-exposure, even though the reason remains uncertain [29,62].

Patients can also be colonised for a long time; however, approximately two-thirds of patients colonised with *C. auris* and discharged to a community setting no longer have detectable *C. auris* colonisation, with no statistically different clinical characteristics between patients that remained positive and the ones that negativised [29]. Nevertheless, patients once colonised remain colonised for months [16,29,62], with skin carriers taking more time to negativise, compared to throat carriers [54]. *C*. *auris*, compared to other *Candida* spp., has the heightened ability to form robust biofilms, colonising the skin, and withstand environmental stresses [79]. It even has the ability to reside in deeper tissues, with decolonisation decreasing the skin burden but not fully eradicating the organism, allowing for fungal regrowth and persistent colonisation [67,80]. On the other hand, without a specific reason identified, when compared to other *Candida* spp., *C. auris* has poor oral colonisation, possibly due to susceptibility to the salivary antimicrobial peptide histatin 5, the lack of key adhesins or host immune factors [68,81], consistent with findings by Alfaifi et al. [30].

Therefore, routine testing should be performed on admission and continued throughout hospitalisation, to exclude nosocomial infection and late or persistent colonisation. This is especially important for patients with frequent readmissions to healthcare facilities, with contact for more than 24 h with an endemic setting in the previous year or who had a previous infection by *C. auris* [29,55,63], highlighting the need for mandatory recording of previous *C. auris* colonisation [33]. Studies also suggest that additional screening should be done on discharge for patients from a *C. auris* endemic setting and on admission to a nonendemic setting if transferred from endemic healthcare facilities or regions [39,55,63].

### 4.4. Who to Screen for C. auris?

Most studies were conducted in general wards and ICUs, and previous contact with the ICU or the hospital was a common exposure. Colonisation rates varied by facility type, being more common in ventilator-capable skilled nursing facilities, compared to those that did not provide care for ventilated patients or any units in a hospital. Long-term care facilities also had a higher prevalence of colonisation [33,35,44,62,65].

There was an increase in *C. auris* after the pandemic, which could be justified by the complexity of the patients, with longer ICU stays and higher ICU mortality, making them more prone to nosocomial infections [50]. This could also be seen in older patients, particularly in the ages of 60–80 years, who were more colonised in the included studies than those below 50 years, which is consistent with reports from other studies [82,83]. This increased susceptibility in older patients could be related to multiple comorbidities, such as congestive heart failure, diabetes mellitus, chronic kidney disease and age-related immune dysfunction, making them susceptible to colonisation and increasing contact with healthcare facilities, particularly frequent hospitalisations [84]. This could also be the reason that St. Maurice et al. showed that mortality rates during hospitalisation in colonised and infected patients were not significantly different, because colonised and infected patients by this yeast have more comorbidities and a higher risk of death [60].

For ages below 60 years, the incidence of *C. auris* may be linked to chronic illnesses and invasive treatments, an event lately occurred more often in this age group [82,85].

All of this highlights the importance of screening for colonisation in the ICU, where patients are generally older, have various comorbidities and will probably be exposed to medical devices such as mechanical ventilation, urinary catheters, drains, be under parental nutrition and antibiotic or antifungal agents. In addition, it is important to screen hospitalised patients, while also collecting detailed information on healthcare exposure prior to admission, particularly after 60 years old [33,84].

Routine screening of healthcare personnel is not supported by the available evidence, as persistent colonisation among staff appears to be uncommon. The principal role of healthcare personnel in transmission prevention is therefore adherence to hand hygiene, appropriate use of personal protective equipment, correct management of shared equipment, and compliance with contact precautions. Staff screening may be considered exceptionally during an outbreak when epidemiological or molecular evidence implicates a healthcare worker as a possible transmission source.

With mortality rates ranging from about 16.7% to 37.7%, and the 90-day mortality rate in colonised patients being significantly higher than in non-colonised patients [44], mortality by this yeast should not be overlooked. Even though most studies did not provide information about the cause of death, the presence of various comorbidities in patients complicates the distinction between the cause of death. Some studies considered that deaths were related to *C. auris* infection [41,51].

Comorbidities that, instead of *C. auris*, could be the cause of death are a confusing factor about colonisation’s mortality, and leave the question of how dangerous this yeast is in terms of mortality. Mortality was higher in older patients, highlighting this confusing factor, but also the association of *C. auris* colonisation and more serious diseases [41]. Comorbidities were present in all colonised or infected patients with *C. auris*, such as exposure to antifungals or antibiotics, mechanical ventilation, urinary catheters, feeding tubes, tracheostomy, CVC and drains, which were all found in different studies to be significantly more common in colonised patients when compared to non-colonised [33,44,53]. A high Candida score also reached statistical significance as a risk factor for the development of infection in two studies [46,54]. Particularly, surgical patients had significantly higher Candida scores than medical patients, even though being a surgical patient was not significant as a risk factor on its own [54]. In Garcia Bustos et al., antifungal therapy, surgical aetiology at ICU admission, arterial catheters, invasive mechanical ventilation and total parenteral nutrition were all independent risk factors for developing infection, with total parenteral nutrition being the foremost risk factor for colonised patients developing infection [46]. Urinary catheterisation was not found to be a risk factor [46]. Infected patients were more likely to have a central line [60]; however, Ferrer Gómez et al. did not find a significant correlation between a central line, parenteral nutrition, or surgical patient and developing infection after colonisation [54].

The significant association of comorbidities and colonisation or infection varies between studies. As a result, patients with these risk factors, such as exposure to antifungals or antibiotics, mechanical ventilation, catheters, parenteral nutrition or feeding tubes, could be at a higher risk of colonisation and developing infection. This information could be important not only for active screening but also for passive screening.

Co-colonisation with other pathogens, such as MDRO, was significantly more common in *C. auris* colonised patients compared to non-colonised [44], with a higher risk of death in *C. auris* co-infection with *SARS-CoV-2*, alone or with MDRO [52]. Carbapenemase-producing organisms (CPO) colonisation or infection, together with recently received healthcare in the Indian subcontinent, were two significant risk factors for colonisation [55]. Co-colonisation being more common in *C. auris* colonised patients shows that this is a group of patients that could benefit from being screened, and presents an opportunity for joining MDRO screenings already in place with the *C. auris* one [72]. The co-detection of other *Candida* species should be interpreted cautiously. In several studies, other species were isolated either concurrently with *C. auris* or during an earlier clinical episode; however, reporting was insufficient to determine whether both organisms were present in the same specimen. Such findings may reflect mixed colonisation, sequential colonisation, or an independent infection caused by another *Candida* species. Consequently, co-isolation should not be interpreted as co-infection without supporting clinical and microbiological evidence, and the contribution of *C. auris* to clinical disease should be assessed according to the sampling site, species-level identification, and the patient’s clinical presentation.

### 4.5. Alignment with International Screening Guidelines

The findings of this review are broadly consistent with international recommendations for risk-stratified screening for *C. auris*. The CDC recommends that screening decisions consider local epidemiology, epidemiological links, facility characteristics and patient-level risk factors, including prolonged or complex healthcare exposure, mechanical ventilation, indwelling devices and colonisation with other multidrug-resistant organisms [66]. ECDC similarly recommends contact screening after identification of a case and consideration of admission screening and pre-emptive isolation for patients transferred from affected healthcare facilities [80]. Current UKHSA guidance supports screening contacts, patients admitted from affected facilities or following overseas healthcare exposure, and all patients in units with ongoing transmission, with periodic active surveillance considered in high-risk settings [86]. The results from this review support these targeted approaches but do not provide sufficient evidence for routine universal screening in facilities with a low expected prevalence and no evidence of transmission.

### 4.6. The Importance of Antifungal Testing

The combination of phenotypic resistance and multiple genomic mutations indicates that *C. auris* is adapting and evolving. Antifungal resistance patterns are observed not only in isolates from patients with invasive infection but also from colonised patients [14]. Of the included studies in this systematic review, only three did not detect resistance to any antifungal agents, underscoring the prevalence of antifungal resistance in this yeast. Resistance to azoles was present in all studies, except one, and resistance to Amphotericin B was variable. Resistance to echinocandins was present in three studies [42,51,58]. In one study, this resistance only appeared in late recurrent episodes, and it was not present in the baseline resistance [51]. In the other, the patient’s prophylactic echinocandin treatment could have selected for resistance [58]. Therefore, it is important to prevent the recurrence of this colonisation through screening and to be aware of the risks of using echinocandins to prevent the emergence of resistance to this antifungal class.

In this review, information about Clades was insufficient to draw conclusions.

However, these resistances were found in isolates from the South African and South Asian Clades, with Taori et al. stating that it was more prevalent in the South African Clade compared to others [42]. The predominance of Clade I in the included studies may partly reflect the geographical distribution of the evidence rather than the global prevalence of this clade. The apparent under-representation of Clades II, IV, V and VI also limits the representativeness of the findings. Broader prospective surveillance incorporating standardised whole-genome sequencing, antifungal susceptibility testing, and clinical metadata is needed to define the relationship between clade, resistance genotype, susceptibility phenotype, transmissibility, and progression from colonisation to invasive infection. Information about mutations associated with antifungal resistance was also sparse; most of them were already known to be related to azole resistance, such as Y132F, mainly, and K143R, V125A, and F126L mutations in the *ERG*11 gene. S639 mutation in the *FKS*1 hotspot-1 mutation, already known to be associated with echinocandin resistance, was present in one study. *ERG*11 encodes lanosterol 14α-demethylase, the target enzyme of azole antifungals. Resistance-associated substitutions can alter the target conformation and reduce azole binding, thereby increasing MIC values. Their effect may be enhanced by additional mechanisms such as TAC1B-mediated regulation of efflux pumps, increased expression or copy number of MDR1, and other clade-associated genomic changes. Direct experimental and clinical evidence links recurrent *C. auris ERG*11 substitutions with reduced azole susceptibility [87]. *FKS*1 encodes the catalytic subunit of β-1,3-D-glucan synthase, which is inhibited by echinocandins. Amino-acid substitutions in recognised Fks1 hotspot regions reduce the sensitivity of glucan synthase to echinocandins, increase MICs, and can produce cross-resistance to anidulafungin, caspofungin, and micafungin [88]. This is clinically important because echinocandins remain the recommended initial therapy for most adults and children over two months with invasive *C. auris* infection.

Rapid detection of established resistance markers can provide an early warning of likely treatment failure, support selection of initial or alternative therapy, and inform public health investigation of resistant lineages. Nevertheless, molecular testing should complement rather than replace phenotypic antifungal susceptibility testing because not every detected variant is functionally associated with resistance, novel resistance mechanisms may be missed, and genotype–phenotype associations are not complete. For a patient who is only colonised, resistance gene detection does not constitute an indication for antifungal treatment.

### 4.7. C. auris and the Skin Microbiome

The importance of the interaction between the microbiome and *C. auris* colonisation is important, with perturbation of the microbiome acting as a risk factor for colonisation and persistence of *C. auris* [53].

In the skin, Malassezia-predominated communities appear resilient to *C. auris* invasion, and *Staphylococcus hominis* may contribute to negative colonisation, while *C. auris*–colonised samples were enriched in *Proteobacteria Proteus mirabilis*, *K. pneumoniae*, *Providencia stuartii* and *Pseudomonas aeruginosa* [53]. As already seen, co-colonisation with other *Candida* spp. was common, in accordance with the literature, which raises the possibility that colonisation and dominance of the microbiome by *Candida* spp. may be an underlying risk factor or precede colonisation by *C. auris* [18]. The question remains whether some of these comorbidities are risk factors for *C. auris* due to changes in the microbiome, as seen in patients in ventilated skilled nursing facilities, which tend to harbour more *Proteobacteria* species, which could be associated with colonisation and transmission [18]. According to the literature, certain comorbidities alter the microbiomes of the gut and respiratory tract. In Zuo et al., *C. auris* was overrepresented in faecal samples of patients with COVID-19 and community-acquired pneumonia, consistent with the dysbiosis found in critically ill patients [14,49]. Endotracheal tubes were shown to alter the microbiome in ICU patients, that become dominated by Proteobacteria, and tracheostomy tubes by *Pseudomonas*, with the composition of the microbiome correlating with the patient’s prognosis [89,90].

Difficulty in eradicating colonisation by this yeast could also be related to *C. auris* dominance in the microbiome being relatively stable once it occurs [53]. All of this shows that alterations in the microbiome, possibly related to different comorbidities, should be taken into consideration when managing colonisation risk. Finding the relation between different comorbidities and changing of microbiome could help explain and understand which of those risk factors could be more interesting for screening. It may also help to learn how to prevent colonisation and improve decolonisation, knowing that decolonisation agents such as chlorhexidine could alter the microbiome and perpetuate colonisation by certain organisms. Nevertheless, more studies are needed to sustain these conclusions [53].

### 4.8. Limitations of the Study

This review has some important limitations that should be noted. First, there was high methodological heterogeneity among the included studies, particularly regarding study design. A significant proportion consisted of case reports, case series, and retrospective descriptive studies, with small sample sizes and conclusions drawn primarily from characteristics frequently observed among affected patients. This diversity, combined with different approaches to data analysis, made it harder to draw robust and generalizable conclusions on specific topics. Furthermore, inconsistent reporting across studies, with information present in some but lacking in others, limited the comparability of some data. The evidence base comprised cohort, case–control, cross-sectional and prevalence studies, descriptive observational reports, case series, and case reports, conducted in markedly different healthcare settings and patient populations. Screening protocols also varied substantially with regard to eligibility criteria, timing and frequency of sampling, anatomical sites screened, diagnostic methods, and definitions of colonisation and infection. These differences precluded quantitative pooling and limited direct comparison of colonisation prevalence and clinical outcomes across studies.

However, the heterogeneity of the studies allowed this review to include studies from all over the globe, with both endemic and nonendemic settings and diverse screening approaches, thereby helping better understand screening possibilities. This also highlights the need for a well-defined protocol to standardise approaches and allow for easier comparisons between studies.

## 5. Conclusions

**Figure 5:**
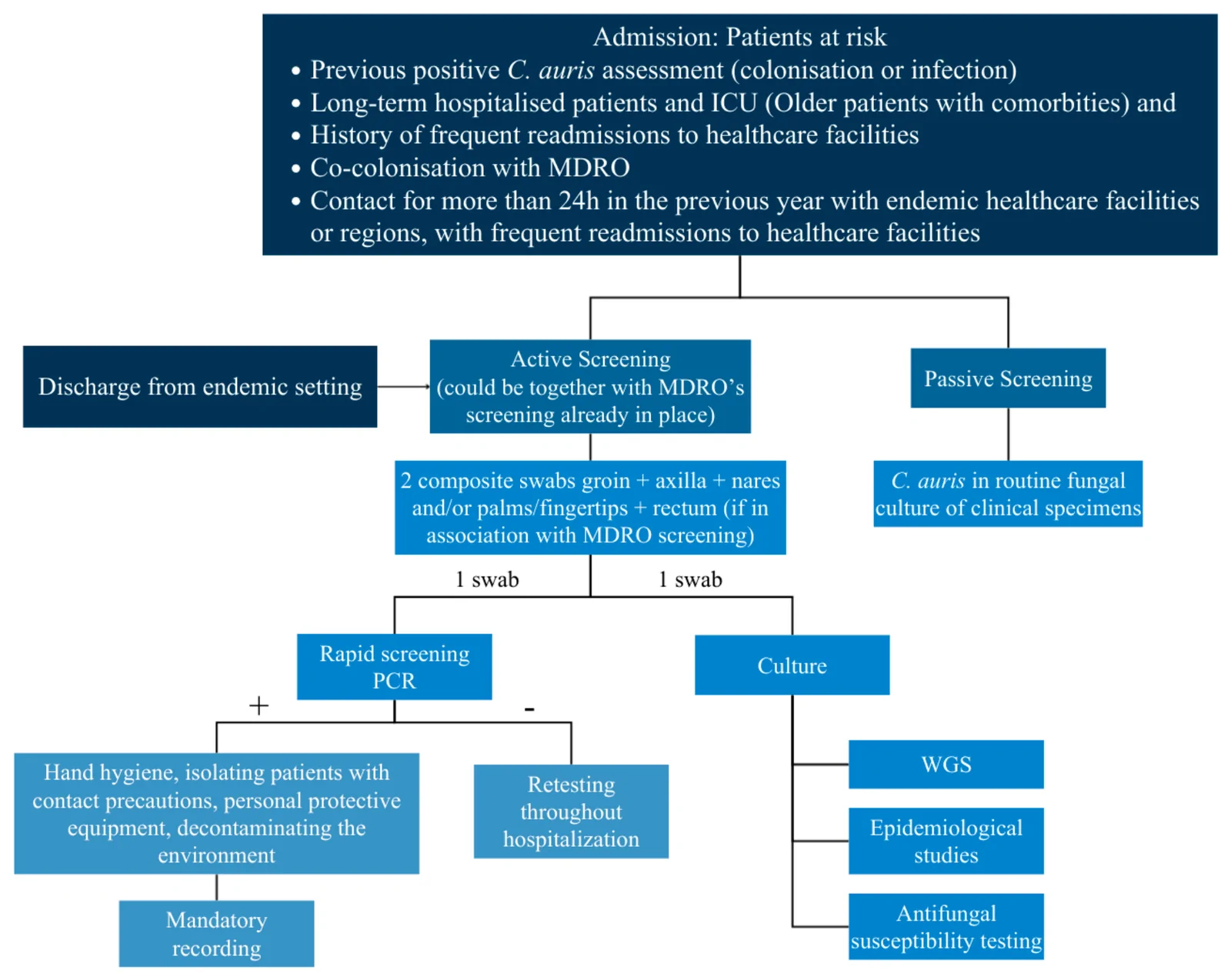
Recommended screening flowchart for *C. auris* colonisation. (ICU: intensive care unit; MDRO: multidrug-resistant organisms).

Based on the results from this systematic review, a recommended flowchart for screening for *C. auris* colonisation is proposed (Figure 5). Therefore, healthcare workers should be on high alert for older patients with comorbidities, mainly long-term hospitalised patients and in ICUs, and patients who are co-colonised with other pathogens. Detailed information on previous health exposure and colonisation status should also be of extreme importance. Testing should start on admission for patients who fall in the following categories: contact for more than 24 h in the previous year with endemic healthcare facilities or regions, frequent readmissions to healthcare facilities, or who had a previous infection by *C. auris*. The screening should be continued regularly throughout hospitalisation. If patients are found to be colonised, this should be recorded to inform future healthcare facilities. On discharge from an endemic setting, patients should also be tested.

Although individual studies identified a comparatively high burden of *C. auris* in the nares or palms/fingertips, the available evidence is insufficient to recommend routine inclusion of these sites in all screening protocols or to support nasal decolonisation as an infection-prevention intervention. Swabs from the rectum could also be used for screening if MDRO screening in this body site is already in place; combine them together. Two swabs should be collected: one for PCR for faster screening, due to its reduced turnaround time and high sensitivity, and another for culture, allowing for epidemiological studies with WGS, providing information about clade, susceptibility and, if relevant, investigating an outbreak, allowing for new studies and information about this ever-increasing yeast.

Treatment of colonisation or decolonisation appears not to have satisfactory results; therefore, they are not advised. Focus should remain on preventing transmission by implementing strategies such as hand hygiene, isolating patients, contact precautions, personal protective equipment, decontaminating the environment and interfacility communication.

Passive surveillance in patients’ routine fungal culture of clinical specimens, if clinically relevant or indicated, should remain a parallel practice with active screening, particularly in samples from urine and the respiratory tract. Active screening comes with logistical hurdles; hence, implementation might not be easy. However, aligning this screening with the ones already in place for MRDO could be a strategy to overcome some of these logistical difficulties. Active screening may initially appear as a disproportionately costly precaution, mainly due to its laboratory costs; however, the financial burden associated with a hospital outbreak, prolonged hospitalisations, high costs of managing multi-drug-resistant infections, frequent failure of antifungal treatments, and the challenge of *C. auris* environmental eradication exceeds the investment in preventive measures [91]. So, in facilities with a high prevalence of colonisation or evidence of ongoing transmission, costs may be offset by earlier implementation of infection-control measures and by preventing downstream expenditure associated with transmission, outbreak investigation, prolonged isolation, environmental decontamination, treatment of invasive infections, and disruption of clinical activity. In low prevalence settings, targeted risk-based screening is likely to be more resource-efficient than indiscriminate universal screening. Formal cost-effectiveness studies conducted across different healthcare systems are required to determine the prevalence thresholds and operational circumstances under which each screening strategy provides the greatest value.

*C. auris* has a high invasive potential in patients and the environment, allowing for rapid growth all over the globe. As a result, a screening protocol alongside well-defined infection-control measures and preparedness should start being implemented to reduce the spread of this multidrug-resistant yeast and allow easier comparisons between different settings.

## Figures and Tables

**Table 1:** Characteristics of the selected studies.

First Author and Year	Country	Study Type	Study Period	Type of Institution	Mean Age of Colonised Patients (Years)	Number of Positive Patients with *C. auris* (Colonisation)	Duration of Hospital Stay Until First *C. auris* Positive Test
Hennessee, 2024 [28]	USA	Retrospective Descriptive Observational	January 2020 to December 2022	Acute care hospitals (10 cases) or long-term acute care hospitals (3 cases) (patients in custody of a state or federal prison)	55 years	11 out of 13 patients with *C. auris*	-
Bergeron, 2021 [29]	USA	Prospective Cohort	October 2017 to February 2019	Community	63 years	36 cases	NA
Alfaifi, 2024 [30]	USA	Retrospective Cross-Sectional	May 2021 to May 2023	Oral Clinic	NA	0 (out of 310 saliva samples tested)	NA
Nascimento, 2024 [31]	Portugal	Prospective Cohort	January 2020 to December 2022	ICU	NA	0	NA
Sharp, 2021 [32]	England	Surveillance Screening	May 2017 and April 2018	ICU	-	0	-
Rowlands, 2023 [33]	USA	Prevalence	November 2017 to November 2019	Ventilator units in two NH (A and B) ICU, CCU, and VPU in one acute care hospital (Hospital C)	Hospital C: 70.4 NHA and NHB: 77.6	188 individuals (6.9%) of 2726 screened admissions Hospital C: 79 (3.6%) positive *C. auris* cases NHA: 67 (20.7%) NHB: 42 (22.0%)	First admission screening within 24 h: 122 (64.9%)
Mesini, 2021 [34]	Italy	Case Report	2021	NICU	25 weeks of gestational age	1	Positive on admission to NICU
Yadav, 2021 [35]	India	Prospective Longitudinal Observational	December 2019 to May 2020	Chronic respiratory diseases’ ward	51	12 (out of 32 patients screened (37.5%))	Admission: 3 (25%) After one week of admission: 9 (75%)
Al-Ramahi, 2023 [36]	Jordan	Case Report	2021	Hospital	48	1	Admission
Arenas, 2024 [37]	USA	Retrospective Cohort	13 December 2021 to 26 July 2022	*C. auris* designated ward	68 years	50	Admission: 31 patients (62%) During hospitalisation: 19
Suárez-Urquiza, 2024 [38]	Spain	Retrospective Cohort	May 2016 to December 2023	ICUs (medical and surgical)	-	Admission: 0.8% of patients were colonised by *C. auris* out of 3206 patients Week four: 16.8%.	ICU admission and subsequently on a weekly basis
Vogelzang, 2019 [39]	Netherlands	Case Series	2019	Hospital	Middle-aged	2	Case 1: not specified Case 2: one day after admission
Steinmann, 2021 [40]	Germany	Case Series	Case 1: August 2019 to September 2020 Case 2: Not specified	Hospital	Middle-aged	2	Case 1: admission Case 2: 285 days into continuous hospitalisation following lung transplantation
Sayeed, 2019 [41]	Pakistan	Retrospective Case Series	September 2014 to March 2017	Hospital (tertiary care referral hospital)	16–35: 5 (18.5%) 36–55: 6 (22.2%) 56–75: 9 (33.3%) 76–95: 7 (25.9%)	27 out of 92 (29.3%)	14 days (colonised and infected)
Taori, 2022 [42]	United Kingdom	Case Series	January 2018 to March 2019	Adult intensive therapy units, diabetes units and cardiac units	30–60: 5 61–70: 5 71–80: 9 >80: 2	21 patients	-
Elbahr, 2024 [43]	Bahrain	Retrospective Cohort	October 2021 to November 2022	Hemato-oncology hospital	61.8	38 cases	-
Rossow, 2021 [44]	USA	Case–Control	August 2016 to September 2018	Ventilator unit in a SNF	68.0 years	60	-
Park, 2024 [45]	UAE	Retrospective Case–Control	October 2019 to June 2023	Hospital	73.5.	66 colonisations and 31 infections (out of 97 positive cultures)	<1 month: 36 (54.5%) 1–3 months: 22 (33.3%) >3 months: 8 (12.1%)
Garcia-Bustos, 2020 [46]	Spain	Retrospective Cohort	March 2016 to March 2019	ICUs (surgical, medical, and burn)	57.75 years	206	-
Levy, 2021 [47]	Reunion Island (France)	Case Series	2010 to 2019	ICU	Case 1: 76 Case 3: 30 Case 4: 83	3	Case 3: Day 30 of hospitalisation
Mirabet, 2021 [48]	Spain	Descriptive Observational Report	Donor 1: April 2017 Donor 2: May 2020	ICU	Donor 1: 55 Donor 2: 34	One donor arteries	7 days (Donor 2)
Zuo, 2020 [49]	China	Prospective Cohort	5 February to 12 May 2020	Hospital	-	*C. auris* were overrepresented in faecal samples of patients with COVID-19 and with community-acquired pneumonia in comparison with healthy controls	-
Magnasco, 2021 [50]	Italy	Retrospective Descriptive Observational	28 February to 31 May 2020	ICU (COVID-19)	63	5 (out of 118 patients in the COVID ICU)	38 days
Briano, 2022 [51]	Italy	Retrospective Cohort	20 February 2020 to 31 May 2021	ICUs (general and neurosurgical critically ill patients ICU, respiratory and COVID-19 ICU, cardiovascular surgical ICU, surgical and solid organ transplant ICU, emergency department ICU)	64 years	157 (out of all the patients admitted to the ICU)	-
Alfonso-Sanchez, 2021 [52]	Spain	Prospective Observational Cohort	27 February 2020 to 26 February 2021	ICU	-	53 (out of 364)	-
Proctor, 2021 [53]	USA	Serial Point Prevalence Survey	January 2019 to April 2019	Ventilator units in a SNF	59	49 (out of 57 residents (86%))	361 days (mean)
Ferrer Gómez, 2021 [54]	Spain	Retrospective Cohort	2018	ICU (anaesthesia)	Skin: 61.18 Throat: 66.83 Skin and throat: 61.21	124 positive patients out of 2130 patients admitted to the ICU	-
Garcia-Jeldes, 2020 [55]	Canada	Cross-Sectional	4 September 2018 to 6 November 2018	Acute care hospitals	-	2 out of 488 patients screened (0.4% overall prevalence) in Canadian patients at higher risk of fungal infections	-
López-Vilella, 2019 [56]	Spain	Case Series	-	Heart Failure and Transplant Unit of the Cardiology Department	61.2 years	5 (4 urgent HT waiting list; 1 post-transplant)	30 days in the ICU
Nobrega de Almeida, 2021 [57]	Brazil	Case–Control	16 December 2020	ICU	64	8 positive cases out of 66 patients at risk of colonisation + index colonised case	26 days (mean)
Woodworth, 2019 [58]	USA	Case Report	Not specified	Hospital	Elderly	1	Several months
Worth, 2020 [59]	Australia	Case Series	2018	Hospital	First case: 70 Contact: 38	2 (first case and contact)	-
De St. Maurice, 2023 [60]	USA	Retrospective Descriptive Observational	1 October 2019 to 28 February 2022	Hospitals	65 years	32 (71%) out of 45 *C. auris* cases	-
Sansom, 2024 [61]	USA	Prospective Cohort	June 2021 to April 2022	vSNFs Acute care hospital	62 years	41 out of 45 patients	-
Pacilli, 2020 [62]	USA	Prevalence	Illinois Surveillance: May 2016 to December 2018 Active Surveillance in Chicago: August 2016 to December 2018 vSNF Chicago Facility Case Study: January to October 2018	Illinois Surveillance: healthcare facility Active Surveillance in Chicago: short-term acute-care hospitals; long-term acute-care hospitals; vSNF; SNF vSNF Chicago Facility Case Study: vSNF	Illinois Surveillance: 63 years (all patients colonised or infected) Active Surveillance in Chicago: not specified vSNF Chicago Facility Case Study: not specified	Illinois Surveillance: 362 Active Surveillance in Chicago (prevalence): Long-term acute-care hospital 31%; vSNF 66%; SNF 2% vSNF Chicago Facility Case Study: 66 out of 114 (58%)	-
Magnasco, 2024 [63]	Italy	Retrospective Descriptive Observational	26 March 2021 to 26 January 2023	ICUs (neurosurgical critically ill patients ICU, respiratory and COVID-19 ICU, cardiovascular surgical ICU, surgical and solid organ transplant ICU, emergency department ICU)	70 (colonised and infected)	76 (out of 84 detected outside the endemic ICU)	18 days (no prior contact with endemic ICU)
Southwick, 2022 [64]	USA	Case Series	26 August 2016 to 7 November 2017	LTCFs, hospitals and long-term acute care facility	74	114 (7% of the 1668)	-
Harris, 2023 [65]	USA	Cross-Sectional	7 March 2023 to 8 June 2023	Acute care hospitals and ventilator-capable LTCFs	-	31 out of 470 patients (6.6%)	-

ICU: intensive care unit; NICU: neonatal intensive care unit; USA: United States of America; NA: not applicable; SNF: skilled nursing facilities; LTCF: long-term care facility; HT: heart transplant; vSNF: ventilator-capable skilled nursing facilities; NH: nursing home; CCU: cardiac care unit; VPU: ventilator/pulmonary unit; UAE: United Arab Emirates.

**Table 2:** Risk factors and clinical outcomes.

First Author and Year	Developed Infection	Co-Infections/Co-Colonisations	Previous Exposures	Death
Hennessee, 2024 [28]	-	-	Mechanical ventilation: 3 Central venous catheters: 2 Urinary catheter: 2 Tracheostomy: 2 Feeding tube: 2 Inpatient medical care at offsite HCF: 12 Inpatient care in an onsite prison medical unit: 1	Yes (1 (9%)) *
Bergeron, 2021 [29]	-	-	Previous positive result for C. auris: 56% (20) screening cases; 44% (16) clinical cases Hospital or Nursing Homes: 100% Surgery: 68% Any indwelling drain, tubes, catheters, or lines during follow-up period: 16/35 (46%) Antibiotic or antifungals during follow-up period: 7/32 (22%)	Yes (13 (36%)—unrelated to *C. auris*)
Alfaifi, 2024 [30]	NA	NA	NA	NA
Nascimento, 2024 [31]	NA	NA	NA	NA
Sharp, 2021 [32]	-	-	-	-
Rowlands, 2023 [33]	-	-	Hospital C: VPU: 5.7%; CCU: 2.5%; ICU: 3.8% Admitted from HCF: 49 (62%) (LTCFs: 65.3%; another unit: 28.6%; another hospital: 6.1%) Admission to a HCF in the 90 days prior: 30 Intubated/tracheostomy: 12/61 (19.7%) Central venous line: 8/49 (16.3%) Drain: 7/62 (11.3%) Antifungal: 4/46 (8.7%) NHA and NHB Drain 49/75 (65.3%) From hospitals: NHA: 98.5%; NHB: 88.1% Another LTCF: NHA: 1.5%; NHB: 7.1% **	-
Mesini, 2021 [34]	No	-	Mother with a vaginal swab positive for *C. auris*	Yes *
Yadav, 2021 [35]	No	*C. albicans*: 2 *C. tropicalis*: 1	Ventilatory support or invasive devices: 0	-
Al-Ramahi, 2023 [36]	No	Carbapenem-resistant *Klebsiella pneumoniae*	Urinary catheter Antibiotics (Azithromycin) Hospitalisation	Yes (unrelated to *C. auris*)
Arenas, 2024 [37]	Yes (3) (BSI)	Colonisation with CRO within 6 months: 12 (24%)	PACFs: 37 (74%) (89% with known ongoing transmission) ICU: 33 (66%) Prior hospitalisation: 28 (56%) Mechanical ventilation: 23 (46%) Tracheostomy: 27 (54%) Urinary catheter: 24 (48%) Central venous catheter: 22 (44%) Feeding tube: 25 (50%) Antibiotics: 28 (56%)	Yes, (10 (20%)) *
Suárez-Urquiza, 2024 [38]	Yes (53/430, 12.32%)—*C. auris* was responsible for 36% of the infections	-	ICU	-
Vogelzang, 2019 [39]	No	Case 1 and 2: Colonised with MDR Gram-negative bacteria (Case 1: OXA-48 and NDM-positive *Enterobacterales*; Case 2: OXA and NDM-positive *Escherichia coli* and NDM-positive *Pseudomonas aeruginosa*—Case 2)	Case 1 and 2: Healthcare exposure in India Case 1: CVC	-
Steinmann, 2021 [40]	No	Case 2: MDRO (*Klebsiella pneumoniae* with extended-spectrum beta-lactamase, vancomycin-resistant *Enterococcus faecium*, carbapenem-resistant *Pseudomonas aeruginosa*). *Candida albicans* (Bronchi); *C. albicans and C. glabrata* (respiratory tract and stool)	Case 1: Urinary catheter; antibiotics; LTCF and hospitalisations Case 2: Antifungal prophylaxis with voriconazole; ICU	Yes (case 2) *
Sayeed, 2019 [41]	-	MDR bacteria: 7 (25.9%)	Surgery: 51.9% ICU: 7 (25.9%) High dependency unit: 6 (22.2%) Ward: 16 (24.6%) Urinary catheter: 17 of 21 urine cases	Crude mortality rate of colonised patients: 33.3%
Taori, 2022 [42]	No	-	Antimicrobial exposure: 20 (95%) Central venous catheter: 10 (48%) Urinary catheter: 9 (43%) Previous hospital stay during an outbreak: 1 Previous stay in wards with *C. auris* colonisation cases: all	Yes (3 (14%)) *
Elbahr, 2024 [43]	Yes (UTI (7); BSI (3); ascitic fluid infection (1); infected bedsore (1))	-	Antifungal: 15 (39.5%)	Day 30 mortality for colonised patients: 23.7% (9/38)
Rossow, 2021 [44]	-	Colonisation or infection with MDROs in the 90 days prior: 26 (43.3%) **	Medical devices: 100% Mechanical ventilation: 53 (88.3%) Urinary catheter: 24 (40%) Tracheostomy: 58 (96.7%) Feeding tube: 56 (93.3%) Antibiotics: 51 (85%) Fluconazole: 1 (11.7%) ≥1 Acute care hospital: 47 (81%) **	90-day all-cause mortality in colonised patients: 16.7% *
Park, 2024 [45]	Yes (16 infected patients had prior colonisation)	MDRO: 24 (36.4%) Multifocal colonisation of other *Candida* spp.: 15 (22.7%) Concomitant bacteriemia: 12 (18.2%)	ICU: 41 (62.1%) Antibacterials: 20 (30.3%) Surgery: 34 (51.5%) Parental nutrition: 9 (13.6%) CVC: 40 (60.6%)/peripherally inserted central catheters: 27 (40.9%) Arterial catheter: 40 (60.6%) Endotracheal/tracheostomy tube: 45 (72.6%)	-
Garcia-Bustos, 2020 [46]	Yes, (37 (21.89%))	MDRO: 98 (47.57%) *Candida* spp. other than *C. auris*: 147 (71.36%)	Antifungal therapy: 95 (46.34%) Surgical aetiology at ICU admission: 118 (57.28%) Arterial catheter 157 (76.21%) Total parenteral nutrition 101 (49.03%) Invasive mechanical ventilation 155 (75.24%) Candida score	-
Levy, 2021 [47]	No	Case 1: *C. tropicalis; Legionella pneumophila* Case 3: *Candida albicans*; *Legionella pneumophila* Case 4: *Candida parapsilosis* and *C. tropicalis*; carbapenem-resistant *Klebsiella pneumoniae*, and vancomycin-resistant *Enterococcus faecium*	Travel/hospitalisation abroad: 2 (Case 1: Mauritius; Case 4: Saudi Arabia and India) Antibiotics: 3 Urinary catheter: 3	No
Mirabet, 2021 [48]	No	Patients: *Candida albicans*, *Citrobacter koseri*, and *Staphylococcus aureus* (Donor 2) Arteries: *Candida albicans* and *C. glabrata*, *Pseudomonas aeruginosa*, Coagulase-negative *Staphylococcus*, *Cutibacterium acnes*	ICU: 100%	Yes (2) *
Zuo, 2020 [49]	-	*C. albicans* and *Aspergillus flavus*	Community-acquired pneumonia COVID-19	-
Magnasco, 2021 [50]	Yes (3) (BSI)	Carbapenem-resistant *Pseudomonas aeruginosa* (4); SARS-CoV-2 (5)	Antibiotics: 4	Yes (2 (40%) of the colonised patients) *
Briano, 2022 [51]	Yes, (27 (17%)) (BSI)	*SARS-CoV-2*: 59% (92)	Hospitalisation: 11 (7%) Surgery: 19 (12%) Antibiotics: 152 (97%) Antifungals: 61 (39%) Invasive mechanical ventilation: 150 (96%) CVC: 157 (100%) Parenteral nutrition: 74 (47%)	30-day mortality for colonisation: 31% (48) 30-day mortality after the onset of *C. auris* candidaemia: 26%
Alfonso-Sanchez, 2021 [52]	-	MDRO 24/364 (6.6%) *SARS-CoV-2* (100%)	ICU	--
Proctor, 2021 [53]	Yes (BSI)	History of carbapenem-resistant bacteria colonisation/infection: 31 (67%) Bacterial species enriched in *C. auris*–colonised samples: *Proteus mirabilis*, *Klebsiella pneumoniae*, *Providencia stuartii*, and *Pseudomonas aeruginosa*	Mechanical ventilation: 23 (50%) Gastrostomy tube: 35(78%) Urinary catheter: 33 (72%) Antibacterials: 34 (74%) **	-
Ferrer Gómez, 2021 [54]	100% (10) of positive blood cultures were carriers	-	High Candida score	Mortality rate for colonised patients: 24.6% (skin), 26.9% (throat)
Garcia-Jeldes, 2020 [55]	-	Colonised/infected with NDM-1 and OXA-48-like CPOs—100%	Travelled and healthcare in India: 100%	-
López-Vilella, 2019 [56]	No	*Klebsiella pneumoniae* (1) Pulmonary aspergillosis (1)	Antibiotic treatment: 100% Micafungin for other *Candida* spp.: 1 (case 2) Central venous: 100% Bladder catheters: 100% Prolonged ICU stays: 100%	Yes (3) *
Nobrega de Almeida, 2021 [57]	-	-	Colonised axillary digital thermometer: 6 **	-
Woodworth, 2019 [58]	No	Carbapenem-resistant *Enterobacteriaceae*	Antifungals (Prophylactic echinocandins) Hospitalisations in India	Yes *
Worth, 2020 [59]	No	-	Health care in UK (first case) and in UAE (contact) Urinary catheter (first case and contact) First case and contact in a common ward	-
De St. Maurice, 2023 [60]	-	Current or prior history of CRE, 2 (6%) Current or prior history of MDRO, 13 (41%) Current or prior history of invasive fungal infections, 1 (3%)	Tracheostomy: 27 (84%) Gastrointestinal tube: 27 (84%) Central line: 7 (29%) Foley catheter: 17 (53%) Chest tube: 1 (3%) LTACH/SNF with known outbreak: 30 (94%)	Yes (5 (16%)) *
Sansom, 2024 [61]	Yes (Urine (1), blood (1), abscess (1))	MDR bacteria: 93% (38/41), (8 ESBL (68%), 25 VRE (61%), 21 MRSA (51%), and 17 CRE (41%))	Invasive devices: >70% with ≥1 device Antibacterial: 16 (36%) Antifungal: 4 (9%) Feeding tube: 30 (67%) Tracheostomy: 14 (31%) Ventilator: 16 (36%) Urinary catheter: 13 (29%)	-
Pacilli, 2020 [62]	Illinois Surveillance: 30 infected patients had prior colonisation Active Surveillance in Chicago: not specified vSNF Chicago Facility Case Study: not specified	Illinois Surveillance: 261 out of 490 (53%) were colonised with a CPO (out of colonised or infected) Active Surveillance in Chicago: not specified vSNF Chicago Facility Case Study: 54 out of 114 were co-colonised with CPO (out of colonised or infected)	Illinois Surveillance: not specified Active Surveillance in Chicago: highest prevalence of *C. auris* colonisation was identified in vSNFs (median prevalence, 66%) and LTACHs (median prevalence, 31%); vSNF Chicago Facility Case Study: Mechanical ventilation	-
Magnasco, 2024 [63]	Yes (3 BSI; 1 Septic arthritis)	-	Endemic ICUs: 61 Wards with patients from the endemic ICU, without prior contact with the ICU: 13 (out of 15)	Overall mortality 20.6% (colonisation and infection)
Southwick, 2022 [64]	Yes, 6 (5%) (BSI)	-	Median of 3 HCF admissions within the 90 days before first positive culture Antibiotics: 49% Mechanical ventilation: 81% Tracheostomy: 80% Percutaneous feeding tube: 70%	30-day (17.5%)/90-day (37.7%) mortality rate (colonisation) 62% deceased in 2-year follow-up period *
Harris, 2023 [65]	-	Colonised with carbapenem-resistant *Acinetobacter baumannii*: 9 (29.0%)	Long-term CFs: 9.3% (18/194) Acute care hospitals: 4.7% (13/276) Mechanical ventilation: 100%	-

* Cause not specified or not known (could have been *C. auris*) ** Only shown previous exposures with statistically significant differences between colonised patients and non-colonised patients NA: not applicable; HCF: healthcare facilities; ICU: intensive care unit; VPU: ventilator/pulmonary unit; CCU: cardiac care unit; LTCF: long-term care facility; LTACH: long-term acute care hospital; SNF: skilled nursing facility; vSNF: ventilator-capable skilled nursing facility; PACF: post acute care facilities; CVC: central venous catheter; NH: nursing home; BSI: bloodstream infection; MDR: multidrug resistant; MDRO: multidrug-resistant organisms; UAE: United Arab Emirates; UK: United Kingdom; UTI: urinary tract infection; CPO: carbapenemase-producing organisms; CRO: carbapenem-resistant organisms; CRE: carbapenem-resistant Enterobacteriaceae; ESBL: extended spectrum beta-lactamase; VRE: vancomycin-resistant Enterococci; MRSA: methicillin-resistant *Staphylococcus aureus*.

**Table 3:** Colonisation sites, sampling and laboratory diagnostics.

First Author and Year	Body Site with Positive Colonisation	Methods Used for Screening	
		To Collect Samples	Identification of *C. auris*
Hennessee, 2024 [28]	-	-	-
Bergeron, 2021 [29]	-	Swabs were collected from the groin, axillae, and previously positive body sites	PCR, culture and MALDI-TOF MS
Alfaifi, 2024 [30]	NA	Saliva samples	Culture: CHROMagar Candida Plus
Nascimento, 2024 [31]	NA	Composite bilateral axillary/inguinal swabs	Culture: Sabouraud Gentamicin Chloramphenicol 2 agar and Candida Chromogenic Medium Identification: MALDI-TOF MS Identify any potential Candida cryptic species: PCR
Sharp, 2021 [32]	-	Swabs from nose; throat; axillae; groin; perineum; rectum; a catheter urine sample. (Individuals)	Culture: Directly onto SDA or CHROMagar followed by SDA Identification: MALDI-TOF MS
Rowlands, 2023 [33]	Axillae/groin swab only: 93 (49.5%) Nares swab only: 32 (17.0%) Both axillae/groin and nares swabs: 61 (32.4%) Unknown source: 2 (1.1%)	Composite swab of bilateral axillae/groin Swab of the bilateral nares	PCR, if positive or inconclusive, conventional culture was performed
Mesini, 2021 [34]	Axillae, skin, eyes and ears	Multiple individual swabs	Cultures followed by MALDI-TOF MS
Yadav, 2021 [35]	Groin: 9 (75%) Nose: 5 (42%) Ear: 4 (33%)	Individual swabs of ear, nose, axillae, groin	Culture: SDA and CHROMagarTM Candida Plus agar; CHROMagarTM Candida medium then SDA plates. Identification: MALDI TOF MS
Al-Ramahi, 2023 [36]	Urine	Individual samples from pressure ulcers and urine	Culture: SDA Identification: Vitek-2 yeast system confirmed with PCR
Arenas, 2024 [37]	Axillae/groin: 50 (100%)	Composite swabs of axillae and groin	PCR
Suárez-Urquiza, 2024 [38]	Predominantly colonised the rectum, followed by axillary, inguinal, oropharyngeal, tracheal/bronchial and urine. PPVs for fungemia: tracheal/bronchial 32.84%, urine 25.44%, rectal 15.75%, oropharyngeal 20.00%, axillary 11.11%, inguinal 10.31%	Individuals’ swabs of oropharynx, axillae, inguinal, rectum Aspirates of urine and tracheal/bronchial regions	Culture: CHROMagar Candida agar Identification: MALDI-TOF
Vogelzang, 2019 [39]	Case 1: Groin and CVC Case 2: Urine	Case 1: Individual samples from axillae, groin, rectum, oropharynx and CVC Case 2: Urine sample	Identification: MALDI-TOF MS (after Sheep blood agar or SDA with chloramphenicol) or CHROMagar
Steinmann, 2021 [40]	Case 1: Wound, urine, several body sites including ear Case 2: Urine	Case 1: Wound, urine, several body sites including ear Case 2: Respiratory tract, stool, urine	Culture: Chromogenic yeast medium Identification: MALDI TOF MS and confirmed by ITS sequencing
Sayeed, 2019 [41]	Asymptomatic candiduria: 21 Central line tip: 4 Ear Swab: 1 Oral Swab: 1	-	Presumptive identification: Combination of resistance to fluconazole, absence of pseudo hyphae on thin Cornmeal Tween80 agar, obtaining profile numbers 2,000,130, 2,000,173, 2,102,173, 6,102,173 on API 20C AUX Verification: D1-D2 sequencing of 28S subunit of rDNA
Taori, 2022 [42]	**-**	Swabs from nose, throat, axillae and groin Urine (catheter)	PCR
Elbahr, 2024 [43]	Postauricular: 6 (15.8%) Inguinal (groin): 23 (60.1%) Axillary: 22 cases (57.8%)	Individual swabs were collected from axillary, groin, and postauricular areas	Culture: SDA Identification: MALDI-TOF
Rossow, 2021 [44]	-	Swab of nares, axillae, and groin	PCR and culture
Park, 2024 [45]	Urine: 37 (56.1%) Respiratory tract: 13 (19.7%) Skin: 10 (15.2%) Other or multiple: 6 (9.1%)	Screening upon ICU admission: Composite swabs of axillae and groin Sample collection: Urine, respiratory tract, ear canal	Culture: SDA (bacterial culture); Chromogenic Candida Agar (screening) Identification: MALDI-TOF MS or VITEK 2 system
Garcia-Bustos, 2020 [46]	Rectum: 172 (83.35%) Urinary tract: 73 (35.44%) Respiratory tract: 65 (31.55%) Oropharyngeal, axillary and other isolates: 113 (54.85%)	Samples from rectum, urine, respiratory tract, skin and oropharynx	Initial Identification: AuxaColor2TM and MALDI-TOF Definitive identification: ITS
Levy, 2021 [47]	Urinary tract: 2 (Case 1 and 4) Skin: 1 (Case 3)	-	Cultures followed by MALDI-TOF MS
Mirabet, 2021 [48]	Patients: Pharyngeal exudate (Donor 2) Tissues: Arterial samples	Patients: Individual nasal, bronchoaspirate, pharyngeal, buccal samples and combined axillary-rectal Tissue: Musculoskeletal tissues, corneas, arteries and skin	Culture: CHROMagar Candida medium Identification: MALDI-TOF MS
Zuo, 2020 [49]	Faecal samples	Faecal samples	PCR
Magnasco, 2021 [50]	BAL: 3 Swabs of the groin, axillary and auricular area: 2	Swabs of the groin, axillary and auricular area Respiratory or urinary samples	Cultures followed by MALDI-TOF MS
Briano, 2022 [51]	Urine: 38 (24%) Respiratory tract: 77 (49%) Skin: 146 (93%)	Composite swabs of axillae and groin Deep respiratory samples (for ventilated patients) Urine cultures	Cultures followed by MALDI-TOF MS
Alfonso-Sanchez, 2021 [52]	-	Samples from nasopharyngeal specimens and urine	Cultures followed by MALDI-TOF MS
Proctor, 2021 [53]	All sites (the highest being nares: 42.9%, palms/fingertips: 40.4%, toe webs: 35.7%)	Individual swabs of anterior nares, external auditory canal, axillae, anterior neck, inguinal crease, perianal skin, toe web, tracheostomy site, combined palm/fingertips swabs and combined buccal mucosa/tongue swabs	Culture: CHROMagar Candida plates and Salt Sabouraud Dulcitol Broth subsequently inoculated onto CHROMagar Candida plates. Identification: MALDI-TOF MS
Ferrer Gómez, 2021 [54]	Skin: 118 Pharyngeal (throat): 52	-	Cultures followed by MALDI-TOF MS
Garcia-Jeldes, 2020 [55]	Axillae/groin swabs	Composite bilateral axillae/groin	Culture: Chromogenic agar and broth pre-enrichment culture Identification: MALDI-TOF Confirmed by WGS
López-Vilella, 2019 [56]	Rectal exudate: 4 Tracheal aspirate: 2 ECMO wound exudate: 1 Nasal exudate: 1 Oropharyngeal exudate: 1	Individual samples from oropharyngeal, rectal, wound and nasal exudates, urine and tracheal aspirate	Culture, MALDI-TOF MS, and definitive DNA ITS regions.
Nobrega de Almeida, 2021 [57]	Axillae: 8 Groins: 5 Nostrils: 3 Ears: 2 CVC: 1	Patients: Individual swabs of axillae, groins, ears, nostrils and pressure ulcers when present. Blood, urine and central venous catheter tip Health care workers: Swabs of skin or trophic nail lesions	Culture: Sabouraud dextrose broth and positive samples were plated on CHROMagar Identification: MALDI- TOF MS
Woodworth, 2019 [58]	Urine from a nephrostomy culture	Urostomy Urine (from the patient’s nephrostomy tube)	Culture: Quantitative standard culture methods Identification: MALDI-TOF
Worth, 2020 [59]	Urine	Composite axillae and groin skin Clinical sites of infection (e.g., wounds, catheter sites)	Culture: Candida chromogenic agar Identification: MALDI-TOF MS
De St. Maurice, 2023 [60]	Axillae and groin	Composite swabs of bilateral axillae and groin	PCR and, if positive, cultured on CHROMagar Passive surveillance: Identified with MALDI-TOF
Sansom, 2024 [61]	Palms/fingertips: 31 (76%) Nares: 29 (71%) Axillae: 23 (56%) Inguinal creases: 23 (56%) Skin: 22 (54%)	Swabs from bilateral nares, axillae, inguinal creases, palms/fingertips, and perianal skin	CHROMagar Candida and Salt Sabouraud Dulcitol Broth and then, if cloudy, onto CHROMagar Candida
Pacilli, 2020 [62]	Bilateral axillaery/inguinal	Composite bilateral axillary/inguinal	Illinois Surveillance: Culture or PCR Active Surveillance in Chicago: Culture: CHROMagar Identification: MALDI-TOF vSNF Chicago Facility Case Study: Culture: CHROMagar Identification: MALDI-TOF
Magnasco, 2024 [63]	Urine: 6 Respiratory tract: 6 Skin: 64	Urine Respiratory sample (mechanically ventilated patients) Composite swabs of bilateral axillae and groin	PCR and Culture on Chromatic Candida and SDA plates (composite swabs) MALDI-TOF MS (composite and urine and respiratory samples)
Southwick, 2022 [64]	81% with axillae/groin and 64% with nasal colonisation;	Nasal swabs Combined axillae and groin swabs	PCR and culture
Harris, 2023 [65]	Axillae/groin	Composite axillae/groin swabs	PCR

Broncho-alveolar lavage (BAL), matrix-assisted laser desorption ionisation-time of flight mass spectrometry (MALDI-TOF MS), polymerase chain reaction (PCR), Sabouraud dextrose agar (SDA), intensive care unit (ICU), not applicable (NA), sequencing of internal transcribed spacer (ITS), whole genome sequencing (WGS), positive predictive value (PPV), central venous catheter (CVC).

**Table 4:** Phylogeny, susceptibility profile and mechanisms of antifungal resistance.

First Author and Year	Clade	AST Methodology/MIC Interpretation	Resistant	Susceptible	Mutations Associated with Drug Resistance
Hennessee, 2024 [28]	-	-	-	-	-
Bergeron, 2021 [29]	-	-	-	-	-
Alfaifi, 2024 [30]	-	-	-	-	-
Nascimento, 2024 [31]	-		-	-	-
Sharp, 2021 [32]	-	-	-	-	-
Rowlands, 2023 [33]	-	-	-	-	-
Mesini, 2021 [34]	-	Sensititre Yeastone/CDC tentative breakpoints	Azoles (Fluconazole, Voriconazole, Posaconazole, Itraconazole)	Amphotericin B Echinocandins (Anidulafungin, Caspofungin, Micafungin)	-
Yadav, 2021 [35]	Clade I	Broth microdilution according to CLSI	Fluconazole (100%) Amphotericin B (37%) Non-susceptible: Voriconazole (25%)	Echinocandins (Micafungin and Anidulafungin)	In *ERG*11 (100%): K143R or Y132F In *TAC*1B: A640V (coexisting with K143R) In *STE*6: K719N 55% of the isolates with two copies of the *MDR*1 gene
Al-Ramahi, 2023 [36]	-	Broth microdilution according to CLSI	No resistance identified	Amphotericin B Azoles (fluconazole, itraconazole, voriconazole and posaconazole) Echinocandins (anidulafungin, caspofungin, micafungin)	-
Arenas, 2024 [37]	-	-	-	-	-
Suárez-Urquiza, 2024 [38]	-	-	-	-	-
Vogelzang, 2019 [39]	Clade I	Broth microdilution according to CLSI/CDC tentative breakpoints	Fluconazole and voriconazole	Amphotericin B, Anidulafungin, And Micafungin	-
Steinmann, 2021 [40]	Clade I	Broth microdilution according to CLSI and EUCAST/EUCAST Ecoffs and CDC tentative breakpoints	Case 1: Fluconazole Case 2: Fluconazole, Voriconazole; Isavuconazole	Case 1: Amphotericin B; Voriconazole, Posaconazole, Isavuconazole, Anidulafungin Case 2: Amphotericin B, Posaconazole, Micafungin, Anidulafungin	In *ERG*11: Y132F
Sayeed, 2019 [41]	-	Broth microdilution according to CLSI	Fluconazole (100%), Voriconazole (28.5%), Amphotericin B (7.9%) (infection and colonisation)	Echinocandins	-
Taori, 2022 [42]	Clade I: 16 Clade III: 3	Vitek 2 and Broth microdilution according to CLSI	Fluconazole; Echinocandins—Anidulafungin (2/3 Clade III); Voriconazole (12/14)	Amphotericin B; Echinocandins—Anidulafungin (Clade I and 1/3 Clade 3); Voriconazole (2/14)	In *ERG*11 Y132F for Clade I and F126L for Clade III
Elbahr, 2024 [43]	-	-	-	-	-
Rossow, 2021 [44]	-	-	-	-	-
Park, 2024 [45]	-	Vitek 2/CDC tentative breakpoints	Fluconazole (78.5%)	Caspofungin (83.3%)	-
Garcia-Bustos, 2020 [46]	-	-	-	-	-
Levy, 2021 [47]	Clade I	Broth microdilution according to EUCAST	Case 4: fluconazole, 5-flucytosine (>64 mg/L), Voriconazole (1)	Case 4: Posaconazole, Amphotericin B, Caspofungin, Micafungin	-
Mirabet, 2021 [48]	-	-	No resistance identified	Amphotericin B (Donor 2)	-
Zuo, 2020 [49]	-	-	-	-	-
Magnasco, 2021 [50]	-	Vitek 2/CDC tentative breakpoints	Amphotericin B Azoles	Echinocandins	-
Briano, 2022 [51]	-	Broth microdilution according to CLSI and Sensititre Yeastone/CDC tentative breakpoints	(Only patients that developed infection) Fluconazole (100%) Amphotericin B (first episodes—56%; late recurrent episodes—14%) Caspofungin (late recurrent episodes—14%)	(Only patients that developed infection) Echinocandins (caspofungin (only in first episode), anidulafungin, micafungin)	-
Alfonso-Sanchez, 2021 [52]	-	CDC tentative breakpoints	Amphotericin B	Azoles and Echinocandins	-
Proctor, 2021 [53]	Clade IV	Sensititre Yeastone/CDC tentative breakpoints	No resistance detected	Fluconazole, Voriconazole, Amphotericin B, Echinocandins (Anidulafungin, Caspofungin, Micafungin)	-
Ferrer Gómez, 2021 [54]	-	Sensititre Yeastone/IDSA cutoffs	Fluconazole and Voriconazole	Amphotericin B Echinocandins (Anidulafungin, Caspofungin, Micafungin)	-
Garcia-Jeldes, 2020 [55]	Clade I	-	-	-	-
López-Vilella, 2019 [56]	-	-	-	-	-
Nobrega de Almeida, 2021 [57]	Clade I	Broth microdilution according to CLSI	No resistance identified	Amphotericin B, Voriconazole, Fluconazole, Anidulafungin	-
Woodworth, 2019 [58]	Clade I	Broth microdilution according to CLSI and Sensititre Yeastone	Fluconazole, Caspofungin	Amphotericin, Flucytosine, And Voriconazole	In *ERG*11: Y132F In *FKS*1 hotspot-1: S639P
Worth, 2020 [59]	Clade I	Broth microdilution	Fluconazole	Caspofungin And Anidulafungin	-
De St. Maurice, 2023 [60]	Clade III	Broth microdilution according to CLSI/CDC tentative breakpoints	Fluconazole, Amphotericin B (11 (28%) of 39 isolates)	Echinocandins (Anidulafungin, Caspofungin, Micafungin)	In *ERG*11 (100% both mutations): V125A and F126L One isolate possessed 2 copies of *ERG*11
Sansom, 2024 [61]	-	-	-	-	-
Pacilli, 2020 [62]	-	-	-	-	-
Magnasco, 2024 [63]	-	-	-	-	-
Southwick, 2022 [64]	-	-	-	-	-
Harris, 2023 [65]	-	-	-	-	-

CDC: Centre for Disease Control and Prevention; CLSI: Clinical and Laboratory Standards Institute; EUCAST: European Committee on Antimicrobial Susceptibility Testing; Ecoff: Epidemiological cut-offs; IDSA: Infectious Diseases Society of America.

## Data Availability

Data are available on reasonable request.

## Supplementary Materials

The following supporting information can be downloaded at: https://www.mdpi.com/article/10.3390/jof12080603/s1, Table S1: PRISMA 2020 checklist; Table S2: Summary of Risk of Bias assessment according to Joanna Briggs Institute checklist by study type.

## Abbreviations

AST	Antifungal susceptibility testing
BAL	Broncho-alveolar lavage
BSI	Bloodstream infection
CCU	Cardiac Care Unit
CDC	Centre for Disease Control and Prevention
CLSI	Clinical and Laboratory Standards Institute
CPO	Carbapenemase-producing organisms
CRE	Carbapenem-resistant Enterobacteriaceae
CRO	Carbapenem-resistant organisms
Ct	Cycle Threshold
CVC	Central Venous Catheter
Ecoff	Epidemiological cut-offs
ECDC	European Centre for Disease Prevention and Control
ESBL	Extended-Spectrum Beta-Lactamase
EU/EEA	European Union/European Economic Area
EUCAST	European Committee on Antimicrobial Susceptibility Testing
HCF	Healthcare facilities
HT	Heart Transplant
ICU	Intensive Care Unit
IDSA	Infectious Diseases Society of America
ITS	Sequencing of internal transcribed spacer
JBI	Joanna Briggs Institute
LTACH	Long-Term Acute Care Hospital
LTCF	Long-Term Care Facility
MALDI-TOF MS	Matrix-assisted laser desorption ionisation-time of flight mass spectrometry
MDR	Multidrug resistant
MDRO	Multidrug-resistant organisms
MICs	Minimum inhibitory concentrations
MRSA	Methicillin-Resistant Staphylococcus aureus
NA	Not Applicable
NH	Nursing Home
NICU	Neonatal Intensive Care Unit
NPV	Negative predictive value
PACF	Post acute care facilities
PCR	Polymerase chain reaction
PPV	Positive predictive value
PRISMA	Preferred Reporting Items for Systematic Reviews and Meta-Analyses
RoB	Risk of bias
SDA	Sabouraud dextrose agar
SNF	Skilled Nursing Facilities
USA	United States of America
UKHSA	United Kingdom Security Agency
UTI	Urinary tract infection
VPU	Ventilator/pulmonary Unit
VRE	Vancomycin-Resistant Enterococci
vSNF	Ventilator-capable Skilled Nursing Facilities
WGS	Whole-genome sequencing
WHO	World Health Organization
