# Supplementary material for: Active Screening for C. auris Colonisation: A Preventive Step or an Excessive Measure? A Systematic Review
Source: J Fungi (Basel). 2026 Aug 13;12(8):603. doi: 10.3390/jof12080603 (PMC13514146; doi:10.3390/jof12080603)
Supplement: Supplementary file 1 [file jof-12-00603-s001.zip › jof-4405063-supplementary.pdf]

Table S1 - PRISMA 2020 Checklist

Section and Topic	Item #	Checklist item	Location where item is reported
**TITLE**			
Title	1	Identify the report as a systematic review.	Page 1
**ABSTRACT**			
Abstract	2	See the PRISMA 2020 for Abstracts checklist.	Page 2
**INTRODUCTION**			
Rationale	3	Describe the rationale for the review in the context of existing knowledge.	Page 3-4
Objectives	4	Provide an explicit statement of the objective(s) or question(s) the review addresses.	Page 4
**METHODS**			
Eligibility criteria	5	Specify the inclusion and exclusion criteria for the review and how studies were grouped for the syntheses.	Page 4, 5
Information sources	6	Specify all databases, registers, websites, organisations, reference lists and other sources searched or consulted to identify studies. Specify the date when each source was last searched or consulted.	Page 5
Search strategy	7	Present the full search strategies for all databases, registers and websites, including any filters and limits used.	Page 5
Selection process	8	Specify the methods used to decide whether a study met the inclusion criteria of the review, including how many reviewers screened each record and each report retrieved, whether they worked independently, and if applicable, details of automation tools used in the process.	Page 5
Data collection process	9	Specify the methods used to collect data from reports, including how many reviewers collected data from each report, whether they worked independently, any processes for obtaining or confirming data from study investigators, and if applicable, details of automation tools used in the process.	Page 5
Data items	10a	List and define all outcomes for which data were sought. Specify whether all results that were compatible with each outcome domain in each study were sought (e.g. for all measures, time points, analyses), and if not, the methods used to decide which results to collect.	Page 5
	10b	List and define all other variables for which data were sought (e.g. participant and intervention characteristics, funding sources). Describe any assumptions made about any missing or unclear information.	Page 6, 7
Study risk of bias assessment	11	Specify the methods used to assess risk of bias in the included studies, including details of the tool(s) used, how many reviewers assessed each study and whether they worked independently, and if applicable, details of automation tools used in the process.	Page 6, 7
Effect measures	12	Specify for each outcome the effect measure(s) (e.g. risk ratio, mean difference) used in the synthesis or presentation of results.	NA
Synthesis methods	13a	Describe the processes used to decide which studies were eligible for each synthesis (e.g. tabulating the study	NA

Section and Topic	Item #	Checklist item	Location where item is reported
		intervention characteristics and comparing against the planned groups for each synthesis (item #5)).	
	13b	Describe any methods required to prepare the data for presentation or synthesis, such as handling of missing summary statistics, or data conversions.	NA
	13c	Describe any methods used to tabulate or visually display results of individual studies and syntheses.	NA
	13d	Describe any methods used to synthesize results and provide a rationale for the choice(s). If meta-analysis was performed, describe the model(s), method(s) to identify the presence and extent of statistical heterogeneity, and software package(s) used.	NA
	13e	Describe any methods used to explore possible causes of heterogeneity among study results (e.g. subgroup analysis, meta-regression).	NA
	13f	Describe any sensitivity analyses conducted to assess robustness of the synthesized results.	NA
Reporting bias assessment	14	Describe any methods used to assess risk of bias due to missing results in a synthesis (arising from reporting biases).	Page 6, 7
Certainty assessment	15	Describe any methods used to assess certainty (or confidence) in the body of evidence for an outcome.	Page 6, 7
**RESULTS**			
Study selection	16a	Describe the results of the search and selection process, from the number of records identified in the search to the number of studies included in the review, ideally using a flow diagram.	Figure 1
	16b	Cite studies that might appear to meet the inclusion criteria, but which were excluded, and explain why they were excluded.	Figure 1
Study characteristics	17	Cite each included study and present its characteristics.	Table 1
Risk of bias in studies	18	Present assessments of risk of bias for each included study.	Page 27, paragraph 4
Results of individual studies	19	For all outcomes, present, for each study: (a) summary statistics for each group (where appropriate) and (b) an effect estimate and its precision (e.g. confidence/credible interval), ideally using structured tables or plots.	NA
Results of syntheses	20a	For each synthesis, briefly summarise the characteristics and risk of bias among contributing studies.	Table 1-4 and S2
	20b	Present results of all statistical syntheses conducted. If meta-analysis was done, present for each the summary estimate and its precision (e.g. confidence/credible interval) and measures of statistical heterogeneity. If comparing groups, describe the direction of the effect.	NA
	20c	Present results of all investigations of possible causes of heterogeneity among study results.	NA
	20d	Present results of all sensitivity analyses conducted to assess the robustness of the synthesized results.	NA
Reporting biases	21	Present assessments of risk of bias due to missing results (arising from reporting biases) for each synthesis assessed.	NA

Section and Topic	Item #	Checklist item	Location where item is reported
Certainty of evidence	22	Present assessments of certainty (or confidence) in the body of evidence for each outcome assessed.	NA
**DISCUSSION**			
Discussion	23a	Provide a general interpretation of the results in the context of other evidence.	Page 27-35
	23b	Discuss any limitations of the evidence included in the review.	Page 35
	23c	Discuss any limitations of the review processes used.	Page 35
	23d	Discuss implications of the results for practice, policy, and future research.	Page 35-37
**OTHER INFORMATION**			
Registration and protocol	24a	Provide registration information for the review, including register name and registration number, or state that the review was not registered.	NA
	24b	Indicate where the review protocol can be accessed, or state that a protocol was not prepared.	NA
	24c	Describe and explain any amendments to information provided at registration or in the protocol.	NA
Support	25	Describe sources of financial or non-financial support for the review, and the role of the funders or sponsors in the review.	Page 37
Competing interests	26	Declare any competing interests of review authors.	Page 37
Availability of data, code and other materials	27	Report which of the following are publicly available and where they can be found: template data collection forms; data extracted from included studies; data used for all analyses; analytic code; any other materials used in the review.	Page 37

From: Page MJ, McKenzie JE, Bossuyt PM, Boutron I, Hoffmann TC, Mulrow CD, et al. The PRISMA 2020 statement: an updated guideline for reporting systematic reviews. BMJ 2021;372:n71. doi: 10.1136/bmj.n71. This work is licensed under CC BY 4.0. To view a copy of this license, visit https://creativecommons.org/licenses/by/4.0/

Table S2 – Summary of Risk of Bias assessment according to Joanna Briggs Institute checklist by study type.

Study	Design	JBI tool	Overall RoB
Magnasco, 2021 [1]	Retrospective descriptive observational	Case series	Moderate
Magnasco, 2024 [2]	Retrospective descriptive observational	Case series	Moderate
Mesini, 2021 [3]	Case report	Case report	Low-Moderate
Park, 2024 [4]	Retrospective case-control	Case-control	Moderate
Proctor, 2021 [5]	Serial point prevalence surveys	Cross-sectional	Moderate
Rossow, 2021 [6]	Case-control	Case-control	Moderate
Yadav, 2021 [7]	Prospective longitudinal observational	Cohort	Moderate
Al-Ramahi, 2023 [8]	Case report	Case report	Low
Alfaifi, 2024 [9]	Retrospective cross-sectional	Prevalence	High
Alfonso-Sanchez, 2021 [10]	Prospective observational cohort	Cohort	Low-Moderate
Arenas, 2024 [11]	Retrospective cohort	Cohort	Moderate
Bergeron, 2021 [12]	Prospective cohort	Cohort	Moderate
Briano, 2022 [13]	Retrospective cohort	Cohort	Low
De St. Maurice, 2023 [14]	Retrospective descriptive observational	Case series	Moderate
Elbahr, 2024 [15]	Retrospective cohort	Cohort	High
Ferrer Gómez, 2021 [16]	Retrospective cohort	Cohort	Moderate
Garcia-Bustos, 2020 [17]	Retrospective cohort	Cohort	Low
Garcia-Jeldes, 2020 [18]	Cross-sectional	Prevalence	Moderate
Harris, 2023 [19]	Cross-sectional	Prevalence	Moderate
Hennessee, 2024 [20]	Retrospective descriptive observational	Case series	Moderate
Suárez-Urquiza, 2024 [21]	Retrospective cohort	Cohort	Moderate

Vogelzang, 2019 [22]	Case series	Case series	Moderate
Levy, 2021 [23]	Case series	Case series	Low–Moderate
López-Vilella, 2019 [24]	Case series	Case series	Moderate
Mirabet, 2021 [25]	Descriptive observational report	Case series	Moderate
Nascimento, 2024 [26]	Prospective cohort	Cohort	Low–Moderate
Nóbrega de Almeida, 2021 [27]	Case-control	Case-control	Moderate
Pacilli, 2020 [28]	Prevalence	Prevalence	Moderate
Rowlands, 2023 [29]	Prevalence	Prevalence	High
Sansom, 2024 [30]	Prospective cohort	Cohort	Low–Moderate
Sayeed, 2019 [31]	Retrospective case series	Case series	Moderate–High
Sharp, 2021 [32]	Surveillance screening	Prevalence	Moderate
Southwick, 2022 [33]	Case series	Case series	Moderate
Steinmann, 2021 [34]	Case series	Case series	Moderate
Taori, 2022 [35]	Case series	Case series	Moderate
Woodworth, 2019 [36]	Case report	Case report	Low–Moderate
Worth, 2020 [37]	Case series	Case series	Moderate
Zuo, 2020 [38]	Prospective cohort	Cohort	Moderate–High

JBI: Joanna Briggs Institute; RoB: Risk of Bias; Low: overall low risk of bias; Moderate: some unclear high impact items; High: Multiple high-impact items negative/unclear.

References

[1] Magnasco L, Mikulska M, Giacobbe DR, Taramasso L, Vena A, Dentone C, et al. Spread of Carbapenem-Resistant Gram-Negatives and *Candida auris* during the COVID-19 Pandemic in Critically Ill Patients: One Step Back in Antimicrobial Stewardship? Microorganisms 2021;9. https://doi.org/10.3390/microorganisms9010095.

[2] Magnasco L, Mikulska M, Sepulcri C, Ullah N, Giacobbe DR, Vena A, et al. Frequency of Detection of *Candida auris* Colonization Outside a Highly Endemic Setting: What Is the Optimal Strategy for Screening of Carriage? JOURNAL OF FUNGI 2024;10. https://doi.org/10.3390/jof10010026.

[3] Mesini A, Saffioti C, Mariani M, Florio A, Medici C, Moscatelli A, et al. First Case of *Candida auris* Colonization in a Preterm, Extremely Low-Birth-Weight Newborn after Vaginal Delivery. JOURNAL OF FUNGI 2021;7. https://doi.org/10.3390/jof7080649.

[4] Park S, Kim H, Hong D, Oh H. *Candida auris*: Understanding the dynamics of *C. auris* infection versus colonization. Med Mycol 2024;62. https://doi.org/10.1093/mmy/myae086.

[5] Proctor DM, Dangana T, Sexton DJ, Fukuda C, Yelin RD, Stanley M, et al. Integrated genomic, epidemiologic investigation of *Candida auris* skin colonization in a skilled nursing facility. Nat Med 2021;27:1401-+. https://doi.org/10.1038/s41591-021-01383-w.

[6] Rossow J, Ostrowsky B, Adams E, Greenko J, McDonald R, Vallabhaneni S, et al. Factors Associated With *Candida auris* Colonization and Transmission in Skilled Nursing Facilities With Ventilator Units, New York, 2016-2018. Clinical Infectious Diseases 2021;72:E753–60. https://doi.org/10.1093/cid/ciaa1462.

[7] Yadav A, Singh A, Wang Y, van Haren MHI, Singh A, de Groot T, et al. Colonisation and Transmission Dynamics of *Candida auris* among Chronic Respiratory Diseases Patients Hospitalised in a Chest Hospital, Delhi, India: A Comparative Analysis of Whole Genome Sequencing and Microsatellite Typing. JOURNAL OF FUNGI 2021;7. https://doi.org/10.3390/jof7020081.

[8] Al-Ramahi JW, Ghanem RA, Sayyouh OH, Al-Jammal D, Said MM, Nasrat S, et al. Report of the First Case of *Candida auris* Identified in Jordan. Microbiol Res (Pavia) 2023;14:1559–67. https://doi.org/10.3390/microbiolres14040107.

[9] Alfaifi A, Brooks JK, Jabra-Rizk MA, Meiller TF, Sultan AS. Does *Candida auris* colonize the oral cavity? A retrospective institutional experience. Oral Dis 2024;30:2716–8. https://doi.org/10.1111/odi.14720.

[10] Alfonso-Sanchez JL, Agurto-Ramirez A, Chong-Valbuena MA, De-Jesús-María I, Julián-Paches P, López-Cerrillo L, et al. The Influence of Infection and Colonization on Outcomes in Inpatients With COVID-19: Are We Forgetting Something? Front Public Health 2021;9. https://doi.org/10.3389/fpubh.2021.747791.

[11] Arenas SP, Persad PJ, Patel S, Parekh DJ, Ferreira TBD, Farinas M, et al. Persistent colonization of *Candida auris* among inpatients rescreened as part of a weekly surveillance program. Infect Control Hosp Epidemiol 2024;45:762–5. https://doi.org/10.1017/ice.2023.251.

[12] Bergeron G, Bloch D, Murray K, Kratz M, Parton H, Ackelsberg J, et al. *Candida auris* Colonization after Discharge to a Community Setting: New York City, 2017-2019. Open Forum Infect Dis 2021;8. https://doi.org/10.1093/ofid/ofaa620.

[13] Briano F, Magnasco L, Sepulcri C, Dettori S, Dentone C, Mikulska M, et al. *Candida auris* Candidemia in Critically Ill, Colonized Patients: Cumulative Incidence and Risk Factors. Infect Dis Ther 2022;11:1149–60. https://doi.org/10.1007/s40121-022-00625-9.

[14] De St. Maurice A, Parti U, Anikst VE, Harper T, Mirasol R, Dayo AJ, et al. Clinical, microbiological, and genomic characteristics of clade-III *Candida auris* colonization and infection in southern California, 2019-2022. Infect Control Hosp Epidemiol 2023;44:1093–101. https://doi.org/10.1017/ice.2022.204.

[15] Elbahr U, Khairy A, Dayyab F, Delos Reyes CS, Pastrana J, Vineeth C, et al. Can daily bathing with 4% chlorhexidine + daily chlorhexidine wipe for 1 week be effective in decolonizing *Candida auris* colonization? European Journal of Clinical Microbiology and Infectious Diseases 2024;43:243–7. https://doi.org/10.1007/s10096-023-04723-5.

[16] Ferrer Gómez C, Solís Albamonte P, Delgado Navarro C, Salvador García C, Tormo Palop N, Andrés Ibáñez JA. Analysis of *Candida auris* candidemia cases in an Intensive Care Unit of a tertiary hospital. Rev Esp Anestesiol Reanim 2021;68:431–6. https://doi.org/10.1016/j.redar.2020.10.013.

[17] Garcia-Bustos V, Salavert M, Ruiz-Gaitán AC, Cabañero-Navalon MD, Sigona-Giangreco IA, Pemán J. A clinical predictive model of candidaemia by *Candida auris* in previously colonized critically ill patients. Clinical Microbiology and Infection 2020;26:1507–13. https://doi.org/10.1016/j.cmi.2020.02.001.

[18] Garcia-Jeldes HF, Mitchell R, McGeer A, Rudnick W, Amaratunga K, Vallabhaneni S, et al. Prevalence of *Candida auris* in Canadian acute care hospitals among at-risk patients, 2018. Antimicrob Resist Infect Control 2020;9. https://doi.org/10.1186/s13756-020-00752-3.

[19] Harris AD, Pineles L, Johnson JK, O'Hara LM, Smith LL, French I, et al. Prevalence of *Acinetobacter baumannii* and *Candida auris* in Patients Receiving Mechanical Ventilation. JAMA, vol. 330, n.d., p. 1769–72. https://doi.org/10.1001/jama.2023.21083.

[20] Hennessee I, Forsberg K, Erskine J, Charles A, Russell B, Reyes J, et al. *Candida auris* in US Correctional Facilities. Emerg Infect Dis 2024;30:S36–40. https://doi.org/10.3201/eid3013.230860.

[21] Suárez-Urquiza P, Pemán J, Gordon M, Favier P, Muñoz-Brell P, López-Hontangas JL, et al. Predicting Fungemia in the ICU: Unveiling the Value of Weekly Fungal Surveillance and Yeast Colonisation Monitoring. J Fungi (Basel) 2024;10. https://doi.org/10.3390/jof10100674.

[22] Vogelzang EH, Weersink AJL, van Mansfeld R, Chow NA, Meis JF, van Dijk K. The First Two Cases of *Candida auris* in The Netherlands. J Fungi (Basel) 2019;5. https://doi.org/10.3390/jof5040091.

[23] Levy Y, Miltgen G, Rousseau A, Lugagne N, Teysseyre L, Traversier N, et al. Case report: Emergence of *candida auris* in the Indian ocean region. American Journal of Tropical Medicine and Hygiene 2021;104:739–43. https://doi.org/10.4269/ajtmh.20-0758.

[24] López-Vilella R, Ruiz-Gaitán AC, Costa JRG, Marqués-Sulé E, Guillén R V, García JP, et al. *Candida auris* and Heart Transplantation. Preoperative Attitude. OBM Transplant 2019;3. https://doi.org/10.21926/obm.transplant.1901053.

[25] Mirabet V, Salvador C, Valentín A, Escobedo-Lucea C, Navarro L, Gimeno C, et al. Risk assessment of arterial allograft contamination from tissue donors colonized by *Candida auris*. Journal of Hospital Infection 2021;112:49–53. https://doi.org/10.1016/j.jhin.2021.03.003.

[26] Nascimento T, Inácio J, Guerreiro D, Patrício P, Proença L, Toscano C, et al. Insights into *Candida* Colonization in Intensive Care Unit Patients: A Prospective Multicenter Study. JOURNAL OF FUNGI 2024;10. https://doi.org/10.3390/jof10060378.

[27] Nobrega de Almeida J, Brandão IB, Francisco EC, de Almeida SLR, de Oliveira Dias P, Pereira FM, et al. Axillary Digital Thermometers uplifted a multidrug-susceptible *Candida auris* outbreak among COVID-19 patients in Brazil. Mycoses 2021;64:1062–72. https://doi.org/10.1111/myc.13320.

[28] Pacilli M, Kerins JL, Clegg WJ, Walblay KA, Adil H, Kemble SK, et al. Regional emergence of *candida auris* in Chicago and lessons learned from intensive follow-up at 1 ventilator-capable skilled nursing facility. Clinical Infectious Diseases 2020;71:E718–25. https://doi.org/10.1093/cid/ciaa435.

[29] Rowlands J, Dufort E, Chaturvedi S, Zhu Y, Quinn M, Bucher C, et al. *Candida auris* admission screening pilot in select units of New York City health care facilities, 2017-2019. Am J Infect Control 2023;51:866–70. https://doi.org/10.1016/j.ajic.2023.01.012.
[30] Sansom SE, Gussin GM, Schoeny M, Singh RD, Adil H, Bell P, et al. Rapid Environmental Contamination with *Candida auris* and Multidrug-Resistant Bacterial Pathogens Near Colonized Patients. Clinical Infectious Diseases 2024;78:1276–84. https://doi.org/10.1093/cid/ciad752.
[31] Sayeed MA, Farooqi J, Jabeen K, Awan S, Mahmood SF. Clinical spectrum and factors impacting outcome of *Candida auris*: A single center study from Pakistan. BMC Infect Dis 2019;19. https://doi.org/10.1186/s12879-019-3999-y.
[32] Sharp A, Muller-Pebody B, Charlett A, Patel B, Gorton R, Lambourne J, et al. Screening for *Candida auris* in patients admitted to eight intensive care units in England, 2017 to 2018. Eurosurveillance 2021;26. https://doi.org/10.2807/1560-7917.ES.2021.26.8.1900730.
[33] Southwick K, Ostrowsky B, Greenko J, Adams E, Lutterloh E, Denis RJ, et al. A description of the first *Candida auris*-colonized individuals in New York State, 2016-2017. Am J Infect Control 2022;50:358–60. https://doi.org/10.1016/j.ajic.2021.10.037.
[34] Steinmann J, Schrauzer T, Kirchhoff L, Meis JF, Rath PM. Two *candida auris* cases in Germany with no recent contact to foreign healthcare—epidemiological and microbiological investigations. JOURNAL OF FUNGI 2021;7. https://doi.org/10.3390/jof7050380.
[35] Taori SK, Rhodes J, Khonyongwa K, Szendroi A, Smith M, Borman AM, et al. First experience of implementing *Candida auris* real-time PCR for surveillance in the UK: detection of multiple introductions with two international clades and improved patient outcomes. Journal of Hospital Infection 2022;127:111–20. https://doi.org/10.1016/j.jhin.2022.06.009.
[36] Woodworth MH, Dynerman D, Crawford ED, Doernberg SB, Ramirez-Avila L, Serpa PH, et al. Sentinel case of *Candida auris* in the Western United States following prolonged occult colonization in a returned traveler from India. Microbial Drug Resistance 2019;25:677–80. https://doi.org/10.1089/mdr.2018.0408.
[37] Worth LJ, Harrison SJ, Dickinson M, van Diemen A, Breen J, Harper S, et al. *Candida auris* in an Australian health care facility: importance of screening high risk patients. Medical Journal of Australia 2020;212:510-511.e1. https://doi.org/10.5694/mja2.50612.
[38] Zuo T, Zhan H, Zhang F, Liu Q, Tso EYK, Lui GCY, et al. Alterations in Fecal Fungal Microbiome of Patients With COVID-19 During Time of Hospitalization until Discharge. Gastroenterology 2020;159:1302-1310.e5. https://doi.org/10.1053/j.gastro.2020.06.048.
